# Prenatal Exposure to Ambient Pesticides and Preterm Birth and Term Low Birthweight in Agricultural Regions of California

**DOI:** 10.3390/toxics6030041

**Published:** 2018-07-21

**Authors:** Chenxiao Ling, Zeyan Liew, Ondine S. von Ehrenstein, Julia E. Heck, Andrew S. Park, Xin Cui, Myles Cockburn, Jun Wu, Beate Ritz

**Affiliations:** 1 Department of Epidemiology, Fielding School of Public Health, UCLA, Los Angeles, CA 90095, USA; lingcx@ucla.edu (C.L.); zeyanliew@ucla.edu (Z.L.); ovehren@ucla.edu (Q.S.v.E.); jeheck@ucla.edu (J.E.H.); apark1986@ucla.edu (A.S.P.); cynthiacui1010@ucla.edu (X.C.); 2 Department of Community Health Sciences, Fielding School of Public Health, UCLA, Los Angeles, CA 90095, USA; 3 Department of Preventive Medicine, Keck School of Medicine, University of Southern California, Los Angeles, CA 90089, USA; myles.cockburn@ucdenver.edu; 4 Department of Epidemiology, Colorado School of Public Health, University of Colorado, Aurora, CO 80045, USA; 5 Colorado Comprehensive Cancer Center, University of Colorado, Aurora, CO 80045, USA; 6 Program in Public Health, Susan and Henry Samueli College of Health Sciences, University of California, Irvine, CA 92697, USA; junwu@uci.edu

**Keywords:** agricultural pesticides, residential proximity, adverse birth outcomes, preterm birth, low birthweight, pregnancy

## Abstract

Findings from studies of prenatal exposure to pesticides and adverse birth outcomes have been equivocal so far. We examined prenatal exposure to agricultural pesticides in relation to preterm birth and term low birthweight, respectively, in children born between 1998 and 2010, randomly selected from California birth records. We estimated residential exposure to agriculturally applied pesticides within 2 km of residential addresses at birth by pregnancy trimester for 17 individual pesticides and three chemical classes (organophosphates, pyrethroids, and carbamates). Among maternal addresses located within 2 km of any agricultural pesticide application, we identified 24,693 preterm and 220,297 term births, and 4412 term low birthweight and 194,732 term normal birthweight infants. First or second trimester exposure to individual pesticides (e.g., glyphosates, paraquat, imidacloprid) or exposure to 2 or more pesticides in the three chemical classes were associated with a small increase (3–7%) in risk for preterm birth; associations were stronger for female offspring. We did not find associations between term low birthweight and exposure to pesticides other than myclobutanil (OR: 1.11; 95% CI: 1.04–1.20) and possibly the pyrethroids class. Our improved exposure assessment revealed that first and second trimester exposure to pesticides is associated with preterm delivery but is rarely linked with term low birthweight.

## 1. Introduction

During the first decade of the 21st century, the rates of preterm birth and low birthweight were 11–13% and 7–8% in the US, respectively [1]. While the survival of infants born preterm and/or low birthweight has improved, these children are at higher risk for adverse health outcomes, such as neurodevelopmental impairment, respiratory and gastrointestinal complications [2,3], obesity, diabetes mellitus, hypertension, kidney disease [4,5,6], and infant and childhood mortality [7]. 

California is the largest agricultural state in the United States, with more than 150 million pesticide active ingredients applied every year [8]. Pesticides have been found in indoor dust at residences near agricultural fields and may persist for years [9]. Experimental studies show that various pesticides, including organophosphates and pyrethroids, can influence prenatal development, including disturbance of placental function [10], endocrine disruption [11], immune regulation, and inflammation [12,13]. 

However, epidemiologic studies examining the effects of pesticides on preterm birth and low birthweight have yielded inconsistent results. While ecological and cross-sectional studies have reported positive associations between preterm birth and low birthweight and pesticide use in agriculture [14,15,16], results from studies assessing self-reported or occupational use of pesticides have been inconsistent [17,18,19]. Nevertheless, small biomarker-based studies with measured organochlorines and/or organophosphates and their metabolic breakdown products in maternal blood, urine, or umbilical cord blood have suggested positive associations with preterm birth or with lower birthweight, with variation between the chemicals and outcomes assessed [20,21,22]. A systematic review of 25 studies of residential proximity to pesticide applications suggested weak or no effects on preterm birth and low birthweight, possibly due to misclassification in the exposure assessment [23]. Yet, more recent residential proximity studies using simple or area-level exposure assessments have provided some evidence that pesticides influence birth outcomes [16,24,25]. Three Geographic Information System (GIS)-based studies of the San Joaquin Valley of California (~1997–2011) reported conflicting results. After summing all chemicals with acute toxicity based on the Signal Word established by the US Environmental Protection Agency (EPA) [26], one study found that pesticide exposure increases the risk of preterm birth and low birthweight among those exposed to the highest fifth percentile applied within a 2.6 km^2^ section [27]. The other two studies assessed ever/never exposure to individual agrochemicals and chemical groups, including endocrine disruptors and reproductive toxicants, applied within 500 m of residences in relation to spontaneous preterm birth [28] or pre-eclampsia phenotypes resulting in preterm delivery [29] for exposure in any month of gestation and—contrary to the first study—reported overwhelmingly inverse associations. These discrepancies in results may be explained by the varying pesticides included in these studies as well as the different methods of pesticide exposure assessment, or the chosen exposure period in relation to the gestational age.

Our objective was to examine whether prenatal exposure to agricultural pesticides contributes to the risk of preterm birth or term low birth weight. We estimated GIS-derived exposure to agricultural pesticides applied near maternal residences during pregnancy, which were selected based on previous research that indicated reproductive toxicity, and we considered trimester-specific exposure windows in all agricultural regions of California (defined as areas with any agricultural pesticide application within 2 km of individuals’ residences).

## 2. Materials and Methods 

### 2.1. Study Population 

We combined two sets of birth records randomly selected from all California births between 1998 and 2010; these were (1) controls matched to children with autism in a 1:10 ratio by sex and birth year (*n* = 339,210), as described previously [30] and (2) controls matched to children diagnosed with cancers in a 1:20 ratio by birth year (*n* = 143,595) [31], which were representative of all California births. We excluded children with missing data for gestational length based on the date of the last reported menses (*n* = 20,124), with extreme or implausible gestational ages (<20 weeks or >45 weeks) or birthweights (<500 g or >6800 g) (*n* = 6390), with missing sex (*n* = 2), with home addresses outside of California (*n* = 1433), as well as multiple births (*n* = 13,251) and also removed duplicate subjects. The remaining births included 41,089 preterm births, defined as having a gestational age less than 37 weeks, and 358,256 term births (not low birthweight) between 37 and 41 weeks 6 days as the reference. To examine term low birthweight, we used 7407 term births with a birthweight of less than 2500 g, indicating intrauterine growth restriction, and included 317,710 term normal birthweight infants (2500–4000 g) in the comparison group. We restricted our study population to those residing at birth within 2 km of fields on which agricultural use pesticides were applied, and we included 24,693 preterm births and 220,297 term births, and 4412 term low birthweight and 194,732 term normal birthweight infants. 

### 2.2. Exposure Assessment 

We geocoded maternal residential addresses listed on the birth certificates using an automated approach [32] and calculated measures of residential ambient pesticide exposure using a GIS-based Residential Ambient Pesticide Estimation System, as previously described [33,34]. In brief, we combined California’s Pesticide Use Reports (PUR), land use maps, and geocoded birth addresses to produce estimates of pesticide exposure during each month of pregnancy (see Supplementary Materials and Methods in Appendix A). Monthly exposure estimates (pounds per acre) were calculated by adding the poundage of pesticide applied in a 2 km buffer surrounding each address and weighting the total poundage by the proportion of acreage treated within the buffer. We defined the first, second, and third trimesters as 0–12 weeks, 13–25 weeks, and ≥26 weeks of pregnancy, respectively. For preterm births, the length of gestation and hence, the exposure period are shorter than those of term birth by design; to account for this, we assessed third trimester exposure at 27–32 weeks of gestation only (>88% of preterm births had a gestational length longer than 32 weeks). For each pesticide, monthly values were divided into the daily poundage for each gestational day of pregnancy which was then averaged across all days in each trimester. Due to the uncertainty in this type of exposure assessment (e.g., assuming the mothers stayed at the reported residences during the entire pregnancy, wind patterns), we categorized prenatal exposure as ever/never exposed to a specific chemical in each trimester. 

We selected 17 individual chemicals previously observed to have reproductive toxicity [24,35,36,37,38,39]. Additionally, we considered all pesticides from three widely used chemical classes in the Pesticide Action Network (PAN) pesticide database (http://www.pesticideinfo.org/) that have been linked to reproductive toxicity [21,37,40,41], i.e., 24 N-methyl carbamate/dithiocarbamates, 50 organophosphates, and 29 pyrethroid pesticides, to which one or more study subjects were exposed according to our 2 km buffer criterion (Table S1). Briefly, for each trimester, we identified whether mothers were ever or never exposed (1 vs. 0) to selected individual chemicals within each chemical class; for each chemical class, we generated a count which we categorized into three levels (exposed to 2 or more pesticides, exposed to 1 pesticide, and no exposure).

Since the specific locations of non-agricultural pesticide applications (structural pest control, rights of way, and landscape maintenance in urban communities) are not provided by the PUR, and due to competing exposures such as air pollution in urban areas [42,43], we restricted our analyses to individuals born in agricultural regions, defined as residences within a 2 km buffer of any type of agricultural pesticide application during pregnancy (Figure S1).

### 2.3. Statistical Analysis

**Figure 1:**
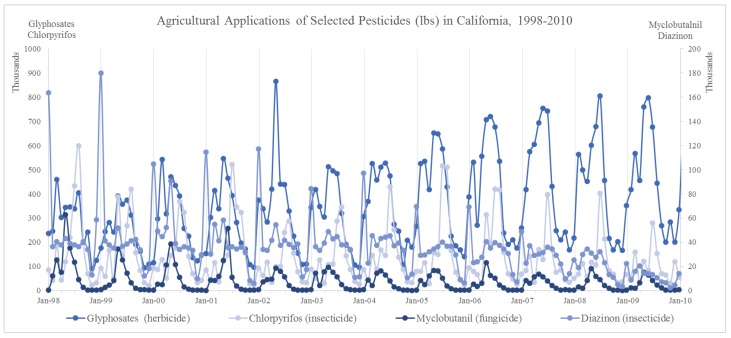
Seasonal variation of selected pesticide applications.

We conducted unconditional logistic regression analyses adjusting for matching factors (infant sex and year of birth) and the source of control subjects (autism vs. cancer study) to estimate the odds ratios (ORs) and 95% confidence intervals (CIs) of associations between pesticide exposure and preterm birth or term low birthweight. To account for the unbalanced sex ratio (~4:1 male: female among the autism controls) and birth year distribution in this combined sample, we included the inverse of the sampling fraction (calculated as the sample size divided by total births in California by gender and birth year) as a stabilized weighting factor to reflect the sex and birth year distribution of all California births. We additionally adjusted for potential confounders based on the literature [44,45,46,47], including maternal age at delivery (≤19, 20–24, 25–29, 30–34, ≥35), maternal race/ethnicity (non-Hispanic White, Hispanic, Black, Asian/Pacific Islander, others), maternal birthplace (US vs. non-US), maternal education (<12 years, 12 years, 13–15 years, ≥16 years), parity (1, 2, ≥3), payment source for prenatal care as a proxy for family income (private/Health Maintenance Organization/Blue Cross Blue Shield vs. MediCal/government/self-pay), prenatal care in the first trimester (yes vs. no), and neighborhood-level socioeconomic status (SES) [48]. Furthermore, we conducted stratified analyses by maternal race/ethnicity (non-Hispanic Whites, US-born Hispanics, and non-US-born Hispanics), since exposure may be higher among Hispanics, especially recent immigrants, who may live close to agricultural fields and have poor housing conditions [49]; by infant sex, because males are more likely to be born preterm [50,51]; and by season of conception (January–March, April–June, July–September, and October–December), estimated from the last menstrual period and length of gestation, because of seasonal variations in pesticide application (Figure 1). We also conducted several sensitivity analyses to evaluate the robustness of our findings, including adjusting for additional confounders, such as maternal cigarette smoking, pre-pregnancy Body Mass Index (BMI), and air pollution; adjusting for co-exposures to other pesticides in the same exposure window or exposures during earlier windows; restricting our analyses to births with a high geocode quality or with spontaneous vaginal deliveries; and choosing alternative weight cut-offs to define intra-uterine growth restriction (IUGR) at term (see Appendix A). Statistical analyses were performed using SAS 9.4 (SAS Institute Inc., Cary, NC, USA).

## 3. Results

Infants born preterm or born term with a low birthweight were more likely to have mothers of younger age, lower education level, lower neighborhood SES, who started prenatal care after the first trimester, and who used Medi-Cal or another government program instead of private insurance. In addition, preterm births were more likely to be a third or later born child, and to have a mother with Hispanic or Black race/ethnic origin. Term low birthweight infants were more likely to be female and a first-born child, born to Black or Asian mothers (Table 1).

Exposure to pesticides in the first and second trimesters was associated with a small increase in risk for preterm birth (ORs: 1.03~1.07) (Table 2) while third trimester exposure did not increase the risk for preterm birth. Effect estimates were slightly stronger in female infants, except for simazine, which showed stronger effects in males, with ORs of 1.06~1.07 (Table S2). The stratified analysis by season of conception suggested that effect estimates were generally stronger when the first or second trimester of pregnancy concurred with the peak season of pesticide application, i.e., in spring, summer, or fall, and higher temperatures might increase the volatility of some of the pesticides (Table S3).

When examining chemical classes, first trimester exposure to carbamates (OR_1st trimester_: 1.04; 95% CI: 1.00–1.08), or pyrethroids (OR_1st trimester_: 1.06; 95% CI: 1.02–1.09) increased the OR for preterm birth in the group exposed to 2 or more chemicals in each class compared with no exposure (Table 3), while second trimester exposure to carbamates, organophosphates, or pyrethroids was associated with small increases (3–6%) in the OR for preterm birth. Exposure in each class resulted—as one would expect—mainly from being exposed to several of the most prevalent chemicals in each class. In sex specific analyses, we did not observe an elevated OR for preterm birth among male infants but observed slightly stronger increases (7–11%) with exposure during the first and second trimesters among female infants (Table 4). Exposure prevalence was highest in infants born to non-US-born Hispanic mothers, followed by US-born Hispanic mothers, and, in general, pesticide ORs were stronger in these two groups; however, we did not observe a single racial/ethnic subgroup with stronger effect estimates across all three chemical classes (Table S4).

Associations between the selected individual pesticides or chemical classes and term low birthweight for each trimester in pregnancy were mostly null. In the multivariable adjusted models, we only estimated increased ORs for second or third trimester exposure to myclobutanil (OR_2nd trimester_: 1.11; 95% CI: 1.03–1.19; OR_3rd trimester_: 1.11; 95% CI: 1.04–1.20 (Table S5); similarly, exposure to the three chemical classes was not associated with term low birthweight in general, except for marginally elevated odds (OR_1st trimester_: 1.05; 95% CI: 0.98, 1.13; OR_2nd trimester_: 1.06; 95% CI: 0.99, 1.13) in infants exposed to two or more pyrethroids (Table S6).

## 4. Discussion

In this large California study, we compared birth outcomes among women living within 2 km of any type of agricultural pesticide application during pregnancy. We found that first and second trimester exposure to selected individual pesticides known or suspected to be reproductive toxicants was associated with a small to moderate size increase in the risk of preterm birth. Early and mid-pregnancy exposure to chemicals in the classes of pyrethroids, which have come into use in more recent years, and possibly, carbamates and organophosphates, was also linked to preterm birth. We found few pesticides, if any, to be associated with term low birthweight, except the pyrethroid class and myclobutanil—this was possibly a chance observation given that we examined 17 individual chemicals. Yet, term low birthweight is rarer than preterm birth; thus we had less statistical power to estimate small effect sizes accurately.

The positive associations with preterm birth are consistent with earlier small biomarker-based studies [21,52]. This is in contrast with much of the epidemiologic literature to date that has presented little evidence for associations of ambient pesticide exposure with preterm birth thus far [23]. Less than a handful of studies conducted in the US have examined associations between environmental exposure to pesticides from agricultural applications and preterm birth and/or low birthweight and also provided month- or trimester-specific estimates [24,27,28,29]. These studies were almost exclusively conducted using California’s PUR system; nevertheless, they differed from each other and our study in terms of study region, exposure assessment methods, specific pesticides, and outcome assessment. Our California-wide study, restricted to those living near actively farmed fields, is most comparable with an earlier study that focused on residents of the San Joaquin Valley and assessed pesticides labeled with EPA signal word toxicity by summing up their active ingredients applied in a 2.6 km^2^ section surrounding maternal residences; this study reported that exposure to all pesticides in the top fifth percentile in pounds was associated with an increased risk of preterm birth and low birthweight by 5–9% overall [27]. However, this method is problematic because (1) the use of total poundage of all pesticides fails to distinguish between high volume but less toxic agents, and low volume but highly toxic agents, and (2) even if the pesticides were assessed according to their known acute toxicity, this may not reflect reproductive toxicity. Therefore, the potential for exposure misclassification and insufficient exploration of the contributions of individual pesticides or pesticide classes with reproductive toxicity limits the results. In contrast to the abovementioned findings, two other studies restricted their samples to all residents of the San Joaquin Valley instead, which included some major cities/towns, and reported overwhelmingly negative associations between 543 specific chemicals and 69 chemical classes (any vs. no), even for reproductive toxicants and endocrine disruptors, and spontaneous preterm deliveries [28] or preeclampsia with preterm delivery [29] in births from 1998–2011. However, these two studies focused mainly on exposure during the month prior to delivery, rather than early or mid-pregnancy, which are critical exposure periods for placenta development and preterm birth [53,54]. Our sensitivity analysis which stratified by season of conception is in line with this as we found an increased risk with exposure in early and not late pregnancy. It is quite possible that results for late pregnancy exposures are affected by a ‘live-birth selection bias’ [55], i.e., that the most susceptible fetuses exposed to pesticides are lost in early pregnancy. The live-birth bias may lead to underestimation of a possible true effect of exposure in early pregnancy and could even create a spurious protective association in late pregnancy, such that only those who are less susceptible to the exposure survive to late pregnancy and these groups may, in general, have a lower risk for these birth outcomes. Moreover, due to the seasonality of pesticide application, those classified as no or low exposure in late pregnancy could have been highly exposed in early pregnancy during the critical period; thus, when estimating the effects of late exposure alone, they may seem protective.

Our study suggests that pesticide exposure affects preterm birth mostly in female children, similar to a Chinese study that found high levels of metabolites of organophosphate pesticides in maternal urine to be associated with duration of gestation only in girls [21]. It has been suggested that exposure to pesticides in early pregnancy triggers more spontaneous abortions of male fetuses [56] or stillbirths in late pregnancy [57], outcomes that were not captured in our study. It is well known that the male fetus is more vulnerable in utero and is at greater risk of fetal demise, with the male-to-female ratio falling from around 120 male conceptions to 105 boys per 100 girls at birth [58]. Some pesticides are endocrine disruptors, such as those in the organophosphate family that mimic sex steroidal action and resemble estrogenic more than androgenic action in fish models [59].

Maternal, placental, and fetal factors are thought to determine the risk of preterm birth and may be affected by prenatal exposure to environmental chemicals [60,61,62,63]. For example, it is known that chlorpyrifos can cross the placenta, possibly affecting fetal growth and development [64]. Mechanisms by which pesticides may affect the risk of preterm birth include interference with immune pathways and inflammation [65], or with metabolic and endocrine regulatory pathways [60,62] and oxidative stress [61]. Early pregnancy exposure may set the stage for preterm delivery by deregulation of critical immunological or metabolic processes at the maternal–fetal interface in early gestation [66]. For example, in-vitro study results suggested that phosmet and chlorpyrifos alter cell viability and induce an inflammatory cytokine profile, indicating that organophosphates may adversely affect trophoblast cells [39].

In general, we observed stronger associations for births among Hispanic mothers, likely because their exposure was higher. According to a recent agricultural survey, about 90% of female farm workers in California are Mexican-born Hispanics [67]; thus, the non-US-born Hispanic mothers may live near fields where they work, making them more likely to be exposed to ambient pesticides when at home. Unfortunately, information on occupations and occupational addresses of the mothers was not available on birth certificates, and therefore, we could not determine workplace exposure.

Fetal growth restriction, the main reason for low birthweight other than preterm birth, can result from transplacental oxygen and nutrient transport, hypoxia, oxidative stress, placental inflammation, and inhibition of placental growth hormones [68]; these mechanisms may be influenced by toxic exposure to organophosphate and carbamate pesticides [69]. Though we did not find much evidence for associations between term low birthweight and many specific pesticide exposures, others however, reported associations between low birthweight (including preterm low weight births) or a decrease in birthweight and some pesticides, including chlorpyrifos and/or diazinon, carbaryl, methyl bromide, as well as with organophosphate and pyrethroid metabolites measured in maternal urine [18,24,36,41,70]. However, it also has been reported that when adjusting for gestational age, associations with low birthweight were attenuated [70]. Our term low birthweight results may have been underpowered, but our findings are in line with previous reports that found exposure to methyl bromide or pyrethroids to be related to reduced birthweight [24,41]. Several effect estimates fell below the null (OR of 1) which might be due to chance, and the upper confidence intervals were very close to 1. Alternatively, these estimates may indicate a well-known selection bias specific to birth outcome studies [55], i.e., the possibility that at high exposures, the fetus is more likely to not be carried to term or even be lost due to fetal demise.

Most previous pesticide and birth outcome studies examining exposure from home/garden or professional use of pesticides relied on parental interviews after birth [19,71]. These studies have been criticized for their potential selection or recall bias, because mothers who have infants with adverse outcomes may be more likely to participate or recall their pesticide exposures. Other studies using job exposure matrices may have been prone to non-differential exposure measurement errors, and often could not distinguish between types of chemicals. Smaller studies were able to employ biomarkers, such as maternal blood or urine collected in pregnancy or umbilical cord blood samples to measure prenatal chemical concentrations (mostly persistent organochlorines and non-persistent organophosphate metabolites) [20,21,22]. The necessarily small size of such pregnancy cohorts limits the number of outcomes and hence, the study power considerably, and they also have to assume that the chemical concentrations measured in bio-samples reflect exposure during multiple gestational windows accurately despite many pesticides having relatively short half-lives, e.g., hours to a few days for organophosphates [72]. Few studies included multiple bio-samples throughout pregnancy.

The GIS-PUR and record linkage studies [27,28,29], including our own, do not suffer from selection bias due to non-response or exposure recall bias that threatens interview-based studies. However, there are many factors that may affect exposure assessment in these studies, including the exposure buffer size, the accuracy of birth addresses for assessing pregnancy period exposure, and assumptions about maternal time activity, such as time spend at home during days when pesticides are applied. Our GIS approach [34] assessed exposure in a smaller geographic area than PUR data alone (because we incorporated land use data to identify the precise location of crops that PUR data reference), and we considered all linkages between residential locations and sources of pesticide reports (that is, if a pesticide use was mentioned but there was no data on land use for related crops, we included the exposure based on PUR data alone). This approach is more comprehensive than others reported to date, and is demonstrably more sensitive than using PUR alone [33]. Women living close to fields may be quite different in terms of other exposures and SES from those living in towns or cities in these study areas. Different from previous California studies, we expanded the study area to California statewide but only included mothers living within 2 km of agricultural pesticide applications, thus restricting our study area to active farming locations and making unmeasured influences of neighborhood SES and water, soil or air pollution more similar. Other unmeasured sources of pesticide exposure include occupational, home and garden use, or dietary exposure to pesticides which may also be more similar in the women we selected, as suggested elsewhere [73].

Our study has some limitations. We assumed that birth addresses reflected the location of mothers over the entirety of pregnancy. A review on residential mobility during pregnancy showed that, on average, 24% (range 14–32%) of mothers move during pregnancy in the US [74]. While most moving distances were short (median <10 km), this may still result in exposure misclassification when using a 2 km exposure buffer. Particularly, Hispanic mothers are more mobile than White mothers [74], increasing the chance of exposure misclassification. In addition, data on the potential confounders maternal smoking and pre-pregnancy BMI, were only available for four out of 13 study years. However, adjustment for these variables did not change our results more than minimally.

In summary, this study found that first and second but not third trimester exposure to almost all pre-selected pesticides known or suspected to be reproductive toxicants was associated with preterm delivery, but only one pesticide (myclobutanil) and perhaps the pyrethroid class was related to term low birthweight. These associations were stronger in female infants, suggesting possible sex specificity for some of these agents or increased vulnerability in male fetuses that results in selective pregnancy loss.

## Figures and Tables

**Table 1:** Demographic and pregnancy characteristics of the study population in agricultural regions, 1998–2010.

Characteristic	Preterm Birth		Term Birth		Term Low Birthweight		Term Normal Birthweight	
	N = 24,693	%	N = 220,297	%	N = 4412	%	N = 194,732	%
**Infant sex**								
Males	18,586	75.3	161,076	73.1	2972	67.4	140,308	72.1
Females	6107	24.7	59,221	26.9	1440	32.6	54,424	27.9
**Year of Birth**								
1998	1393	5.6	12,327	5.6	262	5.9	10,728	5.5
1999	1483	6.0	12,599	5.7	228	5.2	10,944	5.6
2000	1504	6.1	14,190	6.4	293	6.6	12,381	6.4
2001	1661	6.7	14,759	6.7	279	6.3	12,856	6.6
2002	1808	7.3	15,882	7.2	319	7.2	13,846	7.1
2003	2043	8.3	17,993	8.2	365	8.3	15,684	8.1
2004	2127	8.6	18,030	8.2	385	8.7	15,924	8.2
2005	2127	8.6	18,860	8.6	361	8.2	16,824	8.6
2006	2200	8.9	19,797	9.0	409	9.3	17,680	9.1
2007	2440	9.9	20,616	9.4	448	10.2	18,412	9.5
2008	2359	9.6	21,950	10.0	445	10.1	19,696	10.1
2009	2063	8.4	19,155	8.7	349	7.9	17,160	8.8
2010	1485	6.0	14,139	6.4	269	6.1	12,597	6.5
**Maternal Age**								
19 or less	2976	12.1	21,126	9.6	593	13.4	19,711	10.1
20–24	5772	23.4	50,362	22.9	1045	23.7	45,392	23.3
25–29	5922	24.0	59,782	27.1	1139	25.8	52,667	27.0
30–34	5590	22.6	53,719	24.4	938	21.3	46,661	24.0
35 and older	4432	17.9	35,306	16.0	696	15.8	30,299	15.6
Missing	1	0.0	2	0.0	1	0.0	2	0.0
**Maternal Education**								
<12 years	8238	33.4	62,459	28.4	1351	30.6	55,856	28.7
12 years	6955	28.2	58,683	26.6	1249	28.3	51,883	26.6
13–15 years	4949	20.0	45,809	20.8	866	19.6	40,122	20.6
16+ years	3984	16.1	48,252	21.9	827	18.7	42,367	21.8
Missing	567	2.3	5094	2.3	119	2.7	4504	2.3
**Maternal Race/Ethnicity**								
White, non-Hispanic	5919	24.0	64,600	29.3	928	21.0	55,036	28.3
Hispanic, any race	13,801	55.9	116,509	52.9	2278	51.6	103,428	53.1
Black	1691	6.8	9639	4.4	392	8.9	8843	4.5
Asian/PI	2437	9.9	21,570	9.8	587	13.3	20,193	10.4
Other/Refused	845	3.4	7979	3.6	227	5.1	7232	3.7
**Parity**								
1	9319	37.7	84,535	38.4	2238	50.7	76,877	39.5
2	7060	28.6	70,977	32.2	1087	24.6	62,329	32.0
3 or more	8306	33.6	64,736	29.4	1084	24.6	55,484	28.5
Missing	8	0.0	49	0.0	3	0.1	42	0.0
**Prenatal care in first trimester**								
Yes	19,821	80.3	186,741	84.8	3533	80.1	164,640	84.5
No	4580	18.5	32,233	14.6	834	18.9	28,891	14.8
Missing	292	1.2	1323	0.6	45	1.0	1201	0.6
**Payment type of prenatal care**								
Private/ Health Maintenance Organization/Blue Cross Blue Shield	10,731	43.5	110,816	50.3	1961	44.4	96,650	49.6
MediCal/Govt/self-pay	13,591	55.0	108,385	49.2	2401	54.4	97,085	49.9
Missing	371	1.5	1096	0.5	50	1.1	997	0.5
**Maternal birthplace**								
US	13,585	55.0	119,055	54.0	2337	53.0	104,048	53.4
Non-US countries	11,093	44.9	101,169	45.9	2072	47.0	90,618	46.5
Missing	15	0.1	73	0.0	3	0.1	66	0.0
**Quintiles of neighborhood socioeconomic status**								
1 (Lowest)	7157	29.0	55,746	25.3	1254	28.4	49,635	25.5
2	6403	25.9	55,227	25.1	1174	26.6	48,916	25.1
3	5143	20.8	45,378	20.6	883	20.0	39,879	20.5
4	3451	14.0	35,467	16.1	627	14.2	31,215	16.0
5 (Highest)	2525	10.2	28,364	12.9	474	10.7	24,980	12.8
Missing	14	0.1	115	0.1	.	.	107	0.1

**Table 2:** Odds ratios (95% confidence intervals) for trimester exposures to individual pesticides (ever vs. never) and preterm birth.

Pesticide	First Trimester				Second Trimester			
	Preterm Birth *	Term Birth *	OR ^1^	OR ^2^	Preterm Birth *	Term Birth *	OR ^1^	OR ^2^
**Fungicide**								
Myclobutanil	5307 (22.0%)	47,755 (21.6%)	1.02 (0.99, 1.05)	1.02 (0.99, 1.06)	5366 (22.2%)	48,337 (21.9%)	1.02 (0.98, 1.05)	1.02 (0.99, 1.06)
Chlorothalonil	5511 (22.8%)	49,183 (22.3%)	1.03 (1.00, 1.06)	1.02 (0.99, 1.05)	5585 (23.1%)	49,070 (22.2%)	1.05 (1.02, 1.08)	1.04 (1.01, 1.08)
Mancozeb	3600 (14.9%)	32,779 (14.8%)	1.00 (0.96, 1.04)	0.98 (0.95, 1.02)	3588 (14.8%)	33,038 (15.0%)	0.99 (0.95, 1.02)	0.97 (0.94, 1.01)
**Herbicide**								
Glyphosate compounds	14,346 (59.3%)	127,703 (57.8%)	1.07 (1.04, 1.10)	1.05 (1.02, 1.08)	14,295 (59.1%)	127,672 (57.8%)	1.06 (1.03, 1.09)	1.04 (1.01, 1.07)
Paraquat dichloride	3850 (15.9%)	32,073 (14.5%)	1.11 (1.07, 1.16)	1.07 (1.03, 1.11)	3823 (15.8%)	32,009 (14.5%)	1.11 (1.07, 1.15)	1.06 (1.02, 1.10)
Simazine	2613 (10.8%)	23,310 (10.6%)	1.02 (0.98, 1.07)	1.02 (0.97, 1.06)	2684 (11.1%)	22,978 (10.4%)	1.07 (1.03, 1.12)	1.06 (1.02, 1.11)
**Insecticide**								
Chlorpyrifos	8511 (35.2%)	74,414 (33.7%)	1.06 (1.04, 1.10)	1.03 (1.00, 1.06)	8390 (34.7%)	74,037 (33.5%)	1.05 (1.02, 1.08)	1.02 (0.99, 1.05)
Abamectin	7715 (31.9%)	68,819 (31.2%)	1.04 (1.01, 1.07)	1.02 (0.99, 1.05)	7736 (32.0%)	69,606 (31.5%)	1.02 (0.99, 1.05)	1.01 (0.98, 1.04)
Malathion	5696 (23.6%)	51,530 (23.3%)	1.01 (0.98, 1.04)	0.99 (0.96, 1.03)	5715 (23.6%)	51,429 (23.3%)	1.02 (0.99, 1.05)	1.00 (0.97, 1.03)
Imidacloprid	6107 (25.3%)	53,105 (24.0%)	1.07 (1.04, 1.10)	1.06 (1.03, 1.10)	6139 (25.4%)	54,444 (24.6%)	1.04 (1.01, 1.08)	1.04 (1.00, 1.07)
Diazinon	5319 (22.0%)	46,514 (21.1%)	1.05 (1.01, 1.08)	1.02 (0.99, 1.06)	5185 (21.4%)	45,430 (20.6%)	1.05 (1.01, 1.08)	1.02 (0.99, 1.06)
Permethrin	4597 (19.0%)	40,300 (18.2%)	1.05 (1.02, 1.09)	1.03 (1.00, 1.07)	4465 (18.5%)	40,533 (18.3%)	1.01 (0.97, 1.04)	0.99 (0.95, 1.02)
Dimethoate	3223 (13.3%)	27,905 (12.6%)	1.06 (1.02, 1.10)	1.04 (1.00, 1.08)	3216 (13.3%)	27,874 (12.6%)	1.06 (1.02, 1.10)	1.03 (0.99, 1.07)
Methyl bromide	2448 (10.1%)	21,398 (9.7%)	1.04 (1.00, 1.09)	1.05 (1.00, 1.10)	2337 (9.7%)	20,851 (9.4%)	1.02 (0.98, 1.07)	1.01 (0.96, 1.06)
Carbaryl	2241 (9.3%)	20,285 (9.2%)	1.00 (0.96, 1.05)	1.00 (0.96, 1.05)	2150 (8.9%)	20,160 (9.1%)	0.97 (0.92, 1.01)	0.96 (0.92, 1.01)
Phosmet	1154 (4.8%)	9995 (4.5%)	1.05 (0.99, 1.12)	1.01 (0.95, 1.08)	1099 (4.5%)	9875 (4.5%)	1.01 (0.95, 1.08)	0.97 (0.91, 1.04)
Methyl parathion	448 (1.9%)	3660 (1.7%)	1.11 (1.01, 1.23)	1.05 (0.95, 1.17)	402 (1.7%)	3715 (1.7%)	0.98 (0.88, 1.08)	0.91 (0.82, 1.01)

^1^ Adjusted for year of birth, infant sex. ^2^ Adjusted for year of birth, infant sex, maternal age, maternal education, maternal race/ethnicity, parity, prenatal care in first trimester, payment type of prenatal care, maternal birthplace, and neighborhood SES. * Numbers of exposed cases/controls and the percentages in parenthesis; the number used in each model may vary depending on missing values.

**Table 3:** Odds ratios (95% confidence intervals) for trimester exposures to chemical classes and preterm birth.

Chemical Class	First Trimester					Second Trimester				
	Preterm Birth *	Term Birth *	OR ^1^	OR ^2^	OR ^3^	Preterm Birth *	Term Birth *	OR ^1^	OR ^2^	OR ^3^
**No. of carbamates ever exposed to**										
0 (ref.)	15,419 (63.8%)	143,956 (65.2%)				15,343 (63.5%)	143,806 (65.1%)			
1	4519 (18.7%)	40,328 (18.3%)	1.04 (1.01, 1.08)	1.03 (0.99, 1.07)	1.01 (0.98, 1.05)	4604 (19.0%)	40,390 (18.3%)	1.07 (1.03, 1.10)	1.04 (1.01, 1.08)	1.03 (0.99, 1.07)
2+	4237 (17.5%)	36,613 (16.6%)	1.08 (1.04, 1.11)	1.04 (1.00, 1.08)	1.01 (0.97, 1.06)	4227 (17.5%)	36,702 (16.6%)	1.07 (1.04, 1.11)	1.04 (1.00, 1.08)	1.03 (0.98, 1.08)
**No. of organophosphates ever exposed to**										
0 (ref.)	9523 (39.4%)	90,246 (40.9%)				9469 (39.2%)	90,715 (41.1%)			
1	5105 (21.1%)	46,306 (21.0%)	1.04 (1.01, 1.08)	1.01 (0.98, 1.05)	1.00 (0.96, 1.04)	5263 (21.8%)	46,494 (21.0%)	1.08 (1.04, 1.12)	1.06 (1.02, 1.10)	1.04 (1.00, 1.08)
2+	9546 (39.5%)	84,346 (38.2%)	1.07 (1.03, 1.10)	1.02 (0.99, 1.06)	0.98 (0.94, 1.02)	9442 (39.1%)	83,688 (37.9%)	1.07 (1.04, 1.11)	1.03 (1.00, 1.06)	0.99 (0.95, 1.03)
**No. of pyrethroids ever exposed to**										
0 (ref.)	11,938 (49.4%)	112,936 (51.1%)				11,906 (49.3%)	112,617 (51.0%)			
1	4965 (20.5%)	44,681 (20.2%)	1.05 (1.01, 1.09)	1.03 (0.99, 1.06)	1.03 (0.99, 1.07)	4977 (20.6%)	44,247 (20.0%)	1.06 (1.02, 1.10)	1.04 (1.00, 1.08)	1.03 (0.99, 1.07)
2+	7272 (30.1%)	63,281 (28.6%)	1.09 (1.05, 1.12)	1.06 (1.02, 1.09)	1.06 (1.01, 1.11)	7291 (30.2%)	64,034 (29.0%)	1.08 (1.04, 1.11)	1.05 (1.01, 1.08)	1.04 (0.99, 1.08)

^1^ Adjusted for year of birth and infant sex. ^2^ Adjusted for year of birth, infant sex, maternal age, maternal education, maternal race/ethnicity, paternal race, parity, prenatal care in first trimester, payment type for prenatal care, maternal birthplace, and neighborhood SES. ^3^ Adjusted for year of birth, infant sex, maternal age, maternal education, maternal race/ethnicity, paternal race, parity, prenatal care in first trimester, payment type for prenatal care, maternal birthplace, neighborhood SES, and co-exposure to two other chemical classes. * Number of exposed cases/controls and percentages in parenthesis; the number used in each model may vary depending on missing values.

**Table 4:** Odds ratios (95% confidence intervals) for trimester exposures to chemical classes and preterm birth, stratified by infant sex.

Chemical Class	First Trimester				Second Trimester			
	Preterm Birth *	Term Birth *	OR ^1^	OR ^2^	Preterm Birth *	Term Birth *	OR ^1^	OR ^2^
**Males**								
**No. of carbamates ever exposed to**								
0 (ref.)	11,978 (64.4%)	104,716 (64.9%)			11,937 (64.2%)	104,618 (64.8%)		
1	3408 (18.3%)	29,803 (18.5%)	1.00 (0.96, 1.04)	0.99 (0.95, 1.03)	3466 (18.6%)	29,761 (18.4%)	1.02 (0.98, 1.06)	1.01 (0.97, 1.05)
2+	3215 (17.3%)	26,835 (16.6%)	1.05 (1.00, 1.09)	1.01 (0.97, 1.06)	3198 (17.2%)	26,974 (16.7%)	1.04 (1.00, 1.08)	1.00 (0.96, 1.05)
**No. of organophosphates ever exposed to**								
0 (ref.)	7352 (39.5%)	65,519 (40.6%)			7320 (39.4%)	65,953 (40.9%)		
1	3973 (21.4%)	33,934 (21.0%)	1.04 (1.00, 1.09)	1.02 (0.97, 1.06)	4055 (21.8%)	34,148 (21.2%)	1.07 (1.03, 1.11)	1.05 (1.01, 1.09)
2+	7276 (39.1%)	61,901 (38.4%)	1.05 (1.01, 1.08)	1.01 (0.97, 1.04)	7226 (38.8%)	61,253 (38.0%)	1.06 (1.02, 1.10)	1.02 (0.98, 1.06)
**No. of pyrethroids ever exposed to**								
0 (ref.)	9239 (49.7%)	82,215 (51.0%)			9281 (49.9%)	82,055 (50.9%)		
1	3902 (21.0%)	32,699 (20.3%)	1.06 (1.02, 1.10)	1.03 (0.99, 1.08)	3822 (20.5%)	32,618 (20.2%)	1.03 (0.99, 1.08)	1.01 (0.97, 1.06)
2+	5460 (29.4%)	46,440 (28.8%)	1.04 (1.01, 1.08)	1.01 (0.98, 1.05)	5498 (29.6%)	46,681 (28.9%)	1.04 (1.00, 1.08)	1.01 (0.97, 1.05)
**Females**								
**No. of carbamates ever exposed to**								
0 (ref.)	3861 (63.1%)	38,769 (65.5%)			3835 (62.6%)	38,723 (65.4%)		
1	1171 (19.1%)	10,682 (18.0%)	1.09 (1.02, 1.17)	1.08 (1.00, 1.15)	1195 (19.5%)	10,731 (18.1%)	1.12 (1.05, 1.20)	1.09 (1.01, 1.17)
2+	1090 (17.8%)	9783 (16.5%)	1.11 (1.03, 1.19)	1.07 (1.00, 1.15)	1091 (17.8%)	9778 (16.5%)	1.12 (1.04, 1.20)	1.08 (1.01, 1.17)
**No. of organophosphates ever exposed to**								
0 (ref.)	2402 (39.2%)	24,352 (41.1%)			2385 (39.0%)	24,443 (41.3%)		
1	1276 (20.8%)	12,375 (20.9%)	1.04 (0.97, 1.12)	1.01 (0.94, 1.09)	1331 (21.7%)	12,396 (20.9%)	1.10 (1.02, 1.18)	1.07 (1.00, 1.15)
2+	2444 (39.9%)	22,506 (38.0%)	1.09 (1.03, 1.16)	1.04 (0.98, 1.11)	2406 (39.3%)	22,394 (37.8%)	1.09 (1.03, 1.16)	1.04 (0.98, 1.11)
**No. of pyrethroids ever exposed to**								
0 (ref.)	3003 (49.0%)	30,390 (51.3%)			2969 (48.5%)	30,277 (51.1%)		
1	1226 (20.0%)	11,958 (20.2%)	1.04 (0.97, 1.11)	1.02 (0.95, 1.09)	1263 (20.6%)	11,751 (19.8%)	1.09 (1.02, 1.17)	1.08 (1.00, 1.16)
2+	1893 (30.9%)	16,886 (28.5%)	1.14 (1.07, 1.21)	1.11 (1.04, 1.18)	1889 (30.9%)	17,206 (29.0%)	1.12 (1.05, 1.19)	1.09 (1.02, 1.16)

^1^ Adjusted for year of birth. ^2^ Adjusted for year of birth, maternal age, maternal education, maternal race/ethnicity, paternal race, parity, prenatal care in first trimester, payment type for prenatal care, maternal birthplace, and neighborhood SES. * Numbers of exposed cases/controls and percentages in parenthesis; the number used in each model may vary depending on missing values.

## Supplementary Materials

The following are available online at http://www.mdpi.com/2305-6304/6/3/41/s1, Table S1: Individual pesticides included in chemical classes; Table S2: Odds ratios (95% confidence intervals) for trimester exposure to individual pesticides (ever vs. never exposed) and preterm birth, stratified by infant sex; Table S3: Odds ratios (95% confidence intervals) for trimester exposure to individual pesticides (ever vs. never exposed) and preterm birth, stratified by season of conception; Table S4: Odds ratios (95% confidence intervals) for trimester exposure to chemical classes and preterm birth, stratified by maternal race/ethnicity; Table S5: Odds ratios (95% confidence intervals) for trimester exposure to individual pesticides (ever vs. never) and term low birthweight; Table S6: Odds ratios (95% confidence intervals) for trimester exposure to chemical classes and term low birthweight; Table S7: Odds ratios (95% confidence intervals) for trimester exposure to chemical classes and spontaneous preterm birth; Figure S1: Study subjects in agricultural and non-agricultural regions.

## Appendix A

### Appendix A.1. Supplementary Materials and Methods

#### Appendix A.1.1. Exposure Assessment

In brief, since 1974, agricultural pesticide applications for commercial use have been recorded in Pesticide Use Reports (PUR), mandated by the California Department of Pesticide Regulation (CDPR). Each PUR record includes the name of the pesticide’s active ingredient, the poundage applied, the crop type, and the location and date of application. The California Department of Water Resources (CDWR) performs countywide, large-scale surveys of land use and crop cover every 7–10 years. Land use maps increase spatial resolution because they provide more detailed land use geography that allows us to refine the pesticide applications [33]. Previous pesticide studies used different buffer sizes from 500 m [75,76,77,78], half a mile (804.5 m) [79], 1000 m [80], 1250 m [81], 1600 m [40], 5000 m [24], to up to distances of 8000 m [82], depending on the pesticide of interest, landscape, and weather conditions. Thus, the buffer of 2000 m that we chose should have provided a reasonable distance for assessing pesticide applications around residential addresses.

#### Appendix A.1.2. Sensitivity Analyses

In the sensitivity analyses, we compared effect estimates with and without adjustment for two risk factors of adverse birth outcomes—maternal cigarette smoking during pregnancy (yes vs. no) and pre-pregnancy Body Mass Index (BMI), calculated as maternal pre-pregnancy weight divided by maternal height (kg/m^2^) [45,46]—for births in 2007–2010 only, since these variables are only available on birth certificates from 2007 onwards. We also investigated the potential confounding effects of outdoor air pollution that can impact fetal growth during critical periods [43,83,84] among the autism controls only due to data availability. We estimated trimester-specific exposure to local, traffic-derived NOx, PM_2.5_, and CO, including roadways within 1.5 km of subjects’ birth addresses [42,85], i.e., inter-quartile range (IQR)-scaled measurement of NOx as a local traffic marker derived from the CAlifornia LINE source dispersion model (CALINE4) model [86,87]. Additionally, we adjusted for co-exposure to at least one of the other individual chemicals as a single variable (yes/no) when assessing each individual chemical, and we estimated mutually adjusted ORs for the three chemical class exposures during the same exposure window because of their moderate to strong correlations (r ~0.7). When evaluating later trimester exposures, we adjusted for exposure during prior pregnancy periods, because these effect estimates may be altered by earlier exposures [88]. Since birth addresses with a low geocode quality (i.e., at the USPS Zip Code Area centroid level or coarser) due to missing or non-geocodeable fields on the birth certificates (about 12% of all addresses) is likely to introduce spatial exposure misclassification, we excluded those with a geocode quality at the USPS Zip Code Area centroid level or coarser. In preterm birth analysis, we examined spontaneous vaginal deliveries only, excluding medically indicated preterm deliveries (about 35% of all preterm deliveries in our study population) more likely to be due to severe maternal pregnancy complications including pre-eclampsia [89] and gestational diabetes [90,91] and their etiology might differ from spontaneous preterm deliveries. Lastly, we chose alternative cut-offs (2331 g, 2580 g, or 2693 g as the first, third, or fifth percentile, respectively, among all term births in our study population), rather than the standard 2500 g to define term low birthweight and compared them with a reference group with a birthweight between the chosen cut-off and 4000 g.

### Appendix A.2. Supplementary Results

In our sensitivity analyses, the results were similar with additional adjustment for maternal pre-pregnancy BMI and maternal smoking in the years 2007–2010; when using NOx as a marker for traffic-related air pollution; or after restricting to those with high geocoding quality only. For each individual pesticide, adjustment for co-exposure to other pesticides resulted in an attenuation of odds by 2–3%. ORs mutually adjusted for three chemical classes or adjusted for exposure in prior trimesters were mostly unchanged or slightly smaller; the mutually adjusted OR for pyrethroids was the most stable, suggesting a more robust association with pyrethroids (Table 3). ORs were slightly stronger when we restricted our analysis to spontaneous preterm births only for the pyrethroid class (Table S7) but were generally similar for individual chemicals or other chemical classes. The results for term low birthweights using the third and fifth percentiles as cut-offs were very similar to the original results using 2500 g, while the effect sizes for several pesticides (e.g., permethrin, methyl bromide) were slightly increased by using the first percentile as the cut-off, especially in the second and third trimesters. Assuming that children in the first percentile of weight at term are most likely to represent IUGR infants, we may have increased the specificity of our outcomes and would expect this to have improved and increased any true effect estimates. However, since the number of cases was smaller, the confidence intervals widened substantially, and we cannot exclude chance as an alternate explanation.
