# Supplementary material for: Prenatal Exposure to Ambient Pesticides and Preterm Birth and Term Low Birthweight in Agricultural Regions of California
Source: Toxics. 2018 Jul 21;6(3):41. doi: 10.3390/toxics6030041 (PMC6160921; doi:10.3390/toxics6030041)
Supplement: Supplementary file 1 [file toxics-06-00041-s001.pdf]

Supplementary Materials: Prenatal Exposure to Ambient Pesticides and Preterm Birth and Term Low Birthweight in Agricultural Regions of California

Chenxiao Ling, Zeyan Liew, Ondine S. von Ehrenstein, Julia E. Heck, Andrew S. Park, Xin Cui, Myles Cockburn, Jun Wu and Beate Ritz

Table S1. Individual pesticides included in chemical classes.

Chemical Code[a]	Common Name	Use Type
	**Dithiocarbamates/N-Methyl Carbamates (*n* = 24)**	
369	Maneb	Fungicide
616	Metam-Sodium	Fumigant, Herbicide, Fungicide, Microbiocide, Algaecide
589	Thiram	Fungicide
970	Potassium N-Methyldithiocarbamate	Fumigant, Fungicide, Microbiocide, Algaecide, Nematicide
288	Ferbam	Fungicide
417	Nabam	Fungicide, Herbicide
548	Sodium Dimethyl Dithio Carbamate	Fungicide
211	Mancozeb	Fungicide
629	Ziram	Fungicide, Microbiocide, Dog and Cat Repellent
627	Zineb	Fungicide
493	Metiram	Fungicide
383	Methomyl	Insecticide
105	Carbaryl	Insecticide, Plant Growth Regulator, Nematicide
375	Methiocarb	Insecticide, Molluscicide
1910	Oxamyl	Insecticide, Nematicide
575	Aldicarb	Insecticide, Nematicide
111	Formetanate Hydrochloride	Insecticide
106	Carbofuran	Insecticide, Nematicide
2202	Thiodicarb	Molluscicide, Insecticide
1924	Bendiocarb	Insecticide
62	Propoxur	Insecticide
623	Mexacarbate	Insecticide
1875	Pirimicarb	Insecticide

2201	Butoxycarboxim	Insecticide
	**Organophosphates (n = 50)**	
253	Chlorpyrifos	Insecticide, Nematicide
1685	Acephate	Insecticide
367	Malathion	Insecticide
198	Diazinon	Insecticide
216	Dimethoate	Insecticide
1626	Ethephon	Plant Growth Regulator
418	Naled	Insecticide
335	Phosmet	Insecticide
70	Bensulide	Herbicide
382	Oxydemeton-Methyl	Insecticide
1689	Methidathion	Insecticide
314	Azinphos-Methyl	Insecticide
394	Methyl Parathion	Insecticide, Nematicide
230	Disulfoton	Insecticide, Nematicide
90394	Methyl Parathion, Other Related	Insecticide, Nematicide
92739	Nonanoic Acid, Other Related	Insecticide, Nematicide
1857	Fenamiphos	Insecticide, Nematicide
1697	Methamidophos	Insecticide, Breakdown product
478	Phorate	Insecticide, Nematicide
558	Sulfotep	Insecticide
404	Ethoprop	Insecticide, Nematicide
254	Fonofos	Insecticide
190	S,S,S-Tributyl Phosphorotrithioate	Defoliant, Plant Growth Regulator
459	Parathion	Insecticide
90480	Mevinphos, Other Related	Insecticide
480	Mevinphos	Insecticide
2042	Profenofos	Insecticide
165	Coumaphos	Insecticide
90459	Parathion, Other Related	Insecticide, Nematicide
187	Ddvp	Insecticide, Breakdown product, Impurity

90187	Ddvp, Other Related	Insecticide
90482	Phosphamidon, Other Related	Insecticide
482	Phosphamidon	Insecticide
88	Trichlorfon	Insecticide
566	Demeton	Insecticide, Nematicide
268	Ethion	Insecticide
110	Carbophenothion	Insecticide
305	Tetrachlorvinphos	Insecticide
192	Dioxathion	Insecticide
90192	Dioxathion, Other Related	Insecticide
479	Phosalone	Insecticide
1523	Phosacetin	Rodenticide
263	Epn	Insecticide
72	Dicrotophos	Insecticide
90293	Merphos, Other Related	Defoliant, Plant Growth Regulator
293	Merphos	Defoliant, Plant Growth Regulator
2006	Sulprofos	Insecticide
577	Tepp	Insecticide
90577	Tepp, Other Related	Insecticide
517	Ronnel	Insecticide
	**Pyrethroids (*n* = 29)**	
2300	Bifenthrin	Insecticide
2008	Permethrin	Insecticide
2321	Esfenvalerate	Insecticide
2195	Tau-Fluvalinate	Insecticide
2223	Cyfluthrin	Insecticide
2297	Lambda-Cyhalothrin	Insecticide
2234	Fenpropathrin	Insecticide
3866	(S)-Cypermethrin	Insecticide
3010	Deltamethrin	Insecticide
2171	Cypermethrin	Insecticide
3956	Beta-Cyfluthrin	Insecticide

5877	Gamma-Cyhalothrin	Insecticide
2119	Resmethrin	Insecticide
2329	Tralomethrin	Insecticide
92119	Resmethrin, Other Related	Insecticide
5327	Imiprothrin	Insecticide
4038	D-Trans Allethrin	Insecticide
2093	Phenothrin	Insecticide
12	Allethrin	Insecticide
1963	Fenvalerate	Insecticide
1695	Tetramethrin	Insecticide
92093	Phenothrin, Other Related	Insecticide
90012	Allethrin, Other Related	Insecticide
92008	Permethrin, Other Related	Insecticide
2293	D-Allethrin	Insecticide
3985	Prallethrin	Insecticide
92293	D-Allethrin, Other Related	Insecticide
91695	Tetramethrin, Other Related	Insecticide
4039	S-Bioallethrin	Insecticide

[a] Assigned by California Department of Pesticide Regulation.

Table S2. Odds ratios (95% confidence intervals) for trimester exposure to individual pesticides (ever vs never exposed) and preterm birth, stratified by infant sex.

Pesticide	First trimester				Second trimester			
	Preterm Birth*	Term Birth*	OR[1]	OR[2]	Preterm Birth*	Term Birth*	OR[1]	OR[2]
**Males**								
**Fungicide**								
Myclobutanil	3968 (21.3%)	35111 (21.8%)	0.97 (0.94, 1.01)	0.97 (0.93, 1.01)	4072 (21.9%)	35700 (22.1%)	0.99 (0.95, 1.02)	0.99 (0.95, 1.02)
Chlorothalonil	4147 (22.3%)	35912 (22.3%)	1.00 (0.97, 1.04)	0.99 (0.95, 1.03)	4231 (22.7%)	35845 (22.2%)	1.03 (0.99, 1.07)	1.02 (0.99, 1.06)
Mancozeb	2702 (14.5%)	24012 (14.9%)	0.97 (0.93, 1.01)	0.96 (0.92, 1.00)	2735 (14.7%)	24217 (15.0%)	0.98 (0.93, 1.02)	0.97 (0.93, 1.01)
**Herbicide**								
Glyphosate compounds	11006 (59.2%)	93459 (57.9%)	1.05 (1.02, 1.09)	1.04 (1.01, 1.07)	11018 (59.2%)	93553 (58.0%)	1.05 (1.02, 1.09)	1.04 (1.01, 1.07)

Paraquat dichloride	2964 (15.9%)	23751 (14.7%)	1.10 (1.05, 1.14)	1.06 (1.01, 1.10)	2954 (15.9%)	23577 (14.6%)	1.10 (1.06, 1.15)	1.07 (1.02, 1.11)
Simazine	2093 (11.2%)	17086 (10.6%)	1.07 (1.02, 1.12)	1.06 (1.01, 1.12)	2075 (11.2%)	16938 (10.5%)	1.07 (1.02, 1.12)	1.07 (1.02, 1.13)
**Insecticide**								
Chlorpyrifos	6496 (34.9%)	54491 (33.8%)	1.05 (1.02, 1.08)	1.02 (0.98, 1.05)	6432 (34.6%)	54201 (33.6%)	1.04 (1.01, 1.08)	1.01 (0.98, 1.05)
Abamectin	5812 (31.2%)	50314 (31.2%)	1.00 (0.97, 1.04)	0.99 (0.95, 1.02)	5911 (31.8%)	50950 (31.6%)	1.01 (0.98, 1.04)	0.99 (0.96, 1.03)
Malathion	4431 (23.8%)	37826 (23.4%)	1.02 (0.99, 1.06)	1.00 (0.96, 1.04)	4410 (23.7%)	37965 (23.5%)	1.01 (0.97, 1.05)	0.99 (0.96, 1.03)
Imidacloprid	4608 (24.8%)	38937 (24.1%)	1.04 (1.00, 1.07)	1.03 (0.99, 1.06)	4632 (24.9%)	39660 (24.6%)	1.02 (0.98, 1.06)	1.01 (0.97, 1.05)
Diazinon	3949 (21.2%)	34395 (21.3%)	0.99 (0.96, 1.03)	0.97 (0.93, 1.00)	3910 (21.0%)	33315 (20.6%)	1.02 (0.98, 1.06)	0.99 (0.96, 1.03)
Permethrin	3479 (18.7%)	29333 (18.2%)	1.03 (0.99, 1.07)	1.02 (0.98, 1.06)	3415 (18.4%)	29546 (18.3%)	1.00 (0.96, 1.04)	0.98 (0.94, 1.02)
Dimethoate	2432 (13.1%)	20514 (12.7%)	1.03 (0.99, 1.08)	1.01 (0.96, 1.06)	2430 (13.1%)	20431 (12.7%)	1.03 (0.99, 1.08)	1.01 (0.96, 1.05)
Methyl bromide	1876 (10.1%)	15659 (9.7%)	1.04 (0.99, 1.10)	1.04 (0.98, 1.09)	1786 (9.6%)	15162 (9.4%)	1.02 (0.97, 1.08)	1.00 (0.95, 1.06)
Carbaryl	1753 (9.4%)	14966 (9.3%)	1.02 (0.97,1.07)	1.01 (0.95, 1.06)	1651 (8.9%)	14846 (9.2%)	0.96 (0.91, 1.01)	0.95 (0.90, 1.00)
Phosmet	863 (4.6%)	7197 (4.5%)	1.04 (0.97, 1.12)	1.01 (0.94, 1.09)	840 (4.5%)	7200 (4.5%)	1.01 (0.94, 1.09)	0.98 (0.91, 1.06)
Methyl parathion	331 (1.8%)	2688 (1.7%)	1.06 (0.95, 1.19)	1.02 (0.90, 1.15)	301 (1.6%)	2717 (1.7%)	0.95 (0.84, 1.07)	0.91 (0.80, 1.03)
				**Females**				
**Fungicide**								
Myclobutanil	1388 (22.7%)	12718 (21.5%)	1.07 (1.00, 1.14)	1.08 (1.01, 1.15)	1381 (22.6%)	12811 (21.6%)	1.05 (0.99, 1.12)	1.06 (1.00, 1.14)
Chlorothalonil	1431 (23.4%)	13193 (22.3%)	1.06 (1.00, 1.13)	1.06 (0.99, 1.13)	1440 (23.5%)	13157 (22.2%)	1.07 (1.01, 1.14)	1.06 (1.00, 1.14)
Mancozeb	938 (15.3%)	8763 (14.8%)	1.03 (0.96, 1.11)	1.01 (0.93, 1.09)	918 (15.0%)	8827 (14.9%)	1.00 (0.93, 1.08)	0.98 (0.91, 1.06)
**Herbicide**								
Glyphosate compounds	3646 (59.6%)	34175 (57.7%)	1.08 (1.03, 1.14)	1.07 (1.01, 1.13)	3613 (59.0%)	34122 (57.6%)	1.06 (1.01, 1.12)	1.05 (0.99, 1.10)
Paraquat dichloride	975	8477	1.13	1.09	963	8509	1.11	1.05

	(15.9%)	(14.3%)	(1.05, 1.22)	(1.01, 1.17)	(15.7%)	(14.4%)	(1.03, 1.20)	(0.98, 1.14)
Simazine	630 (10.3%)	6228 (10.5%)	0.97 (0.89, 1.06)	0.96 (0.88, 1.05)	676 (11.0%)	6103 (10.3%)	1.07 (0.98, 1.16)	1.05 (0.96, 1.15)
**Insecticide**								
Chlorpyrifos	2175 (35.5%)	19902 (33.6%)	1.08 (1.02, 1.14)	1.05 (0.99, 1.11)	2134 (34.9%)	19807 (33.4%)	1.06 (1.00, 1.12)	1.02 (0.96, 1.08)
Abamectin	2002 (32.7%)	18436 (31.1%)	1.07 (1.02, 1.14)	1.06 (1.00, 1.12)	1975 (32.3%)	18624 (31.4%)	1.04 (0.98, 1.10)	1.02 (0.96, 1.08)
Malathion	1424 (23.3%)	13746 (23.2%)	1.00 (0.94, 1.06)	0.99 (0.93, 1.05)	1442 (23.6%)	13638 (23.0%)	1.03 (0.97, 1.09)	1.01 (0.95, 1.08)
Imidacloprid	1581 (25.8%)	14184 (23.9%)	1.11 (1.05, 1.18)	1.11 (1.04, 1.18)	1590 (26.0%)	14641 (24.7%)	1.07 (1.01, 1.14)	1.07 (1.01, 1.14)
Diazinon	1402 (22.9%)	12313 (20.8%)	1.12 (1.05, 1.19)	1.10 (1.03, 1.17)	1344 (21.9%)	12132 (20.5%)	1.08 (1.01, 1.15)	1.05 (0.98, 1.12)
Permethrin	1186 (19.4%)	10846 (18.3%)	1.07 (1.00, 1.14)	1.05 (0.98, 1.13)	1139 (18.6%)	10892 (18.4%)	1.01 (0.95, 1.08)	0.99 (0.93, 1.07)
Dimethoate	834 (13.6%)	7433 (12.5%)	1.09 (1.01, 1.18)	1.08 (1.00, 1.17)	832 (13.6%)	7447 (12.6%)	1.08 (1.00, 1.17)	1.06 (0.98, 1.15)
Methyl bromide	623 (10.2%)	5727 (9.7%)	1.05 (0.96, 1.14)	1.06 (0.97, 1.16)	596 (9.7%)	5617 (9.5%)	1.02 (0.93, 1.11)	1.02 (0.93, 1.12)
Carbaryl	557 (9.1%)	5383 (9.1%)	0.99 (0.90, 1.08)	1.00 (0.91, 1.10)	545 (8.9%)	5360 (9.0%)	0.97 (0.89, 1.07)	0.98 (0.90, 1.08)
Phosmet	302 (4.9%)	2720 (4.6%)	1.07 (0.95, 1.21)	1.01 (0.89, 1.15)	281 (4.6%)	2653 (4.5%)	1.01 (0.89, 1.15)	0.96 (0.84, 1.09)
Methyl parathion	119 (1.9%)	976 (1.6%)	1.17 (0.96, 1.41)	1.09 (0.90, 1.33)	105 (1.7%)	995 (1.7%)	1.01 (0.82, 1.23)	0.91 (0.73, 1.12)

[1] Adjusted for year of birth. [2] Adjusted for year of birth, maternal age, maternal education, maternal race/ethnicity, parity, prenatal care in first trimester, payment type of prenatal care, maternal birthplace, and neighborhood SES. * Numbers of exposed cases/controls and the percentages in the parenthesis; numbers used in each model may vary depending on missing values.

Table S3. Odds ratios (95% confidence intervals) for trimester exposure to individual pesticides (ever vs never exposed) and preterm birth, stratified by season of conception.

**Pesticide**	**First trimester**				**Second trimester**			
	**Preterm Birth***	**Term Birth***	**OR[1]**	**OR[2]**	**Preterm Birth***	**Term Birth***	**OR[1]**	**OR[2]**
				**Winter (Jan-Mar)**				
**Fungicide**								
Myclobutanil	1533 (26.3%)	13798 (25.5%)	1.04 (0.98, 1.10)	1.03 (0.96, 1.10)	1541 (26.4%)	14130 (26.1%)	1.01 (0.95, 1.08)	1.02 (0.95, 1.08)

Chlorothalonil	1456 (24.9%)	13363 (24.7%)	1.01 (0.95, 1.08)	1.00 (0.94, 1.07)	1213 (20.8%)	11253 (20.8%)	1.00 (0.93, 1.07)	1.01 (0.94, 1.08)
Mancozeb	1072 (18.4%)	9782 (18.1%)	1.02 (0.95, 1.10)	1.00 (0.93, 1.08)	863 (14.8%)	8084 (14.9%)	0.99 (0.92, 1.07)	0.99 (0.91, 1.07)
**Herbicide**								
Glyphosate compounds	3698 (63.4%)	33953 (62.8%)	1.03 (0.97, 1.09)	1.01 (0.95, 1.07)	3423 (58.7%)	31421 (58.1%)	1.02 (0.97, 1.08)	1.01 (0.95, 1.07)
Paraquat dichloride	969 (16.6%)	8738 (16.2%)	1.03 (0.96, 1.11)	1.00 (0.92, 1.07)	778 (13.3%)	7075 (13.1%)	1.02 (0.94, 1.11)	0.98 (0.90, 1.07)
Simazine	772 (13.2%)	6939 (12.8%)	1.03 (0.95, 1.12)	1.02 (0.94, 1.10)	305 (5.2%)	2519 (4.7%)	1.13 (1.00, 1.27)	1.07 (0.94, 1.22)
**Insecticide**								
Chlorpyrifos	1992 (34.1%)	17604 (32.5%)	1.07 (1.01, 1.13)	1.04 (0.98, 1.11)	2158 (37.0%)	19469 (36.0%)	1.04 (0.98, 1.10)	1.01 (0.95, 1.07)
Abamectin	2045 (35.1%)	18437 (34.1%)	1.04 (0.98, 1.10)	1.02 (0.96, 1.08)	2267 (38.8%)	20613 (38.1%)	1.03 (0.98, 1.09)	1.01 (0.95, 1.07)
Malathion	1412 (24.2%)	13379 (24.7%)	0.97 (0.91, 1.03)	0.94 (0.88, 1.01)	1521 (26.1%)	14003 (25.9%)	1.01 (0.95, 1.07)	0.98 (0.92, 1.05)
Imidacloprid	1411 (24.2%)	12672 (23.4%)	1.04 (0.98, 1.11)	1.04 (0.97, 1.11)	1784 (30.6%)	16136 (29.8%)	1.04 (0.98, 1.10)	1.03 (0.97, 1.09)
Diazinon	1272 (21.8%)	11689 (21.6%)	1.01 (0.94, 1.08)	0.98 (0.92, 1.05)	1239 (21.2%)	11145 (20.6%)	1.04 (0.97, 1.11)	1.02 (0.95, 1.09)
Permethrin	1059 (18.2%)	9726 (18.0%)	1.01 (0.94, 1.09)	0.99 (0.92, 1.06)	1237 (21.2%)	11416 (21.1%)	1.01 (0.94, 1.07)	0.98 (0.92, 1.05)
Dimethoate	708 (12.1%)	6529 (12.1%)	1.00 (0.92, 1.09)	0.99 (0.91, 1.08)	1033 (17.7%)	9206 (17.0%)	1.05 (0.97, 1.12)	1.03 (0.96, 1.11)
Methyl bromide	316 (5.4%)	3055 (5.6%)	0.96 (0.85, 1.08)	0.97 (0.86, 1.10)	605 (10.4%)	5614 (10.4%)	1.00 (0.91, 1.09)	1.00 (0.91, 1.10)
Carbaryl	532 (9.1%)	5048 (9.3%)	0.97 (0.89, 1.07)	0.96 (0.87, 1.06)	634 (10.9%)	6100 (11.3%)	0.96 (0.88, 1.05)	0.96 (0.88, 1.05)
Phosmet	316 (5.4%)	3083 (5.7%)	0.95 (0.84, 1.07)	0.91 (0.80, 1.03)	477 (8.2%)	4380 (8.1%)	1.01 (0.91, 1.11)	0.97 (0.88, 1.08)
Methyl parathion	90 (1.5%)	863 (1.6%)	0.95 (0.77, 1.19)	0.90 (0.71, 1.13)	191 (3.3%)	1945 (3.6%)	0.90 (0.77, 1.05)	0.83 (0.71, 0.98)
**Spring (Apr-Jun)**								
**Fungicide**								
Myclobutanil	1618 (27.6%)	14129 (26.7%)	1.04 (0.98, 1.11)	1.05 (0.99, 1.12)	1118 (19.1%)	9910 (18.7%)	1.02 (0.95, 1.09)	1.03 (0.96, 1.10)
Chlorothalonil	1299 (22.2%)	11184 (21.1%)	1.06 (0.99, 1.13)	1.05 (0.98, 1.12)	1286 (21.9%)	11219 (21.2%)	1.04 (0.97, 1.11)	1.03 (0.96, 1.10)

Mancozeb	894 (15.2%)	8089 (15.3%)	0.99 (0.92, 1.07)	0.98 (0.91, 1.06)	745 (12.7%)	6719 (12.7%)	1.00 (0.92, 1.08)	0.98 (0.91, 1.07)
**Herbicide**								
Glyphosate compounds	3588 (61.2%)	31115 (58.8%)	1.10 (1.04, 1.17)	1.09 (1.03, 1.15)	3207 (54.7%)	28071 (53.1%)	1.07 (1.01, 1.13)	1.05 (0.99, 1.11)
Paraquat dichloride	847 (14.4%)	6861 (13.0%)	1.13 (1.05, 1.23)	1.09 (1.01, 1.18)	834 (14.2%)	6638 (12.5%)	1.16 (1.07, 1.25)	1.11 (1.02, 1.20)
Simazine	329 (5.6%)	2647 (5.0%)	1.12 (1.00, 1.26)	1.11 (0.98, 1.26)	524 (8.9%)	4332 (8.2%)	1.10 (1.00, 1.21)	1.08 (0.98, 1.19)
**Insecticide**								
Chlorpyrifos	2279 (38.9%)	19731 (37.3%)	1.07 (1.01, 1.13)	1.03 (0.98, 1.10)	2162 (36.9%)	19042 (36.0%)	1.04 (0.98, 1.10)	1.01 (0.95, 1.07)
Abamectin	2321 (39.6%)	20118 (38.0%)	1.07 (1.01, 1.13)	1.05 (0.99, 1.11)	1786 (30.5%)	15309 (28.9%)	1.08 (1.01, 1.14)	1.06 (1.00, 1.12)
Malathion	1587 (27.1%)	14264 (27.0%)	1.00 (0.94, 1.06)	1.00 (0.94, 1.06)	1403 (23.9%)	12449 (23.5%)	1.02 (0.96, 1.09)	1.01 (0.94, 1.07)
Imidacloprid	1852 (31.6%)	15613 (29.5%)	1.10 (1.04, 1.17)	1.10 (1.03, 1.17)	1614 (27.5%)	13970 (26.4%)	1.06 (1.00, 1.13)	1.05 (0.99, 1.12)
Diazinon	1383 (23.6%)	11967 (22.6%)	1.05 (0.98, 1.12)	1.04 (0.97, 1.11)	1156 (19.7%)	10336 (19.5%)	1.01 (0.94, 1.08)	1.00 (0.93, 1.07)
Permethrin	1291 (22.0%)	11403 (21.6%)	1.03 (0.96, 1.10)	1.00 (0.93, 1.07)	1113 (19.0%)	10167 (19.2%)	0.99 (0.92, 1.06)	0.97 (0.90, 1.04)
Dimethoate	1074 (18.3%)	9434 (17.8%)	1.03 (0.96, 1.10)	1.00 (0.93, 1.08)	849 (14.5%)	7199 (13.6%)	1.07 (0.99, 1.16)	1.04 (0.96, 1.12)
Methyl bromide	720 (12.3%)	6101 (11.5%)	1.07 (0.99, 1.17)	1.08 (0.99, 1.18)	921 (15.7%)	7947 (15.0%)	1.05 (0.97, 1.13)	1.04 (0.96, 1.12)
Carbaryl	718 (12.3%)	6474 (12.2%)	1.00 (0.92, 1.08)	1.00 (0.91, 1.08)	584 (10.0%)	5211 (9.9%)	1.01 (0.92, 1.10)	1.00 (0.91, 1.10)
Phosmet	567 (9.7%)	4480 (8.5%)	1.16 (1.06, 1.27)	1.13 (1.03, 1.24)	231 (3.9%)	1781 (3.4%)	1.17 (1.02, 1.35)	1.13 (0.98, 1.31)
Methyl parathion	241 (4.1%)	1958 (3.7%)	1.11 (0.96, 1.27)	1.05 (0.92, 1.21)	103 (1.8%)	838 (1.6%)	1.11 (0.90, 1.36)	1.01 (0.82, 1.26)
**Summer (Jul-Sep)**								
**Fungicide**								
Myclobutanil	1137 (18.6%)	10129 (18.6%)	1.00 (0.93, 1.07)	1.01 (0.94, 1.09)	1066 (17.5%)	8842 (16.3%)	1.08 (1.01, 1.16)	1.10 (1.02, 1.18)
Chlorothalonil	1277 (21.0%)	11513 (21.2%)	0.98 (0.92, 1.05)	0.98 (0.91, 1.04)	1392 (22.8%)	11913 (21.9%)	1.05 (0.99, 1.12)	1.03 (0.97, 1.10)
Mancozeb	788 (12.9%)	6815 (12.5%)	1.03 (0.95, 1.11)	1.01 (0.93, 1.10)	838 (13.8%)	7409 (13.6%)	1.00 (0.93, 1.08)	0.98 (0.91, 1.06)

**Herbicide**								
Glyphosate compounds	3348 (54.9%)	28488 (52.4%)	1.11 (1.05, 1.17)	1.08 (1.02, 1.14)	3591 (58.9%)	30903 (56.8%)	1.09 (1.03, 1.15)	1.07 (1.01, 1.13)
Paraquat dichloride	887 (14.6%)	6810 (12.5%)	1.19 (1.10, 1.28)	1.14 (1.05, 1.23)	1122 (18.4%)	8805 (16.2%)	1.17 (1.09, 1.25)	1.12 (1.04, 1.20)
Simazine	518 (8.5%)	4466 (8.2%)	1.03 (0.93, 1.13)	1.00 (0.90, 1.10)	1012 (16.6%)	8416 (15.5%)	1.08 (1.00, 1.16)	1.06 (0.99, 1.14)
**Insecticide**								
Chlorpyrifos	2348 (38.5%)	19375 (35.6%)	1.13 (1.07, 1.19)	1.08 (1.02, 1.14)	1917 (31.4%)	15985 (29.4%)	1.10 (1.04, 1.16)	1.05 (0.99, 1.12)
Abamectin	1805 (29.6%)	15720 (28.9%)	1.04 (0.98, 1.10)	1.02 (0.96, 1.08)	1496 (24.5%)	13166 (24.2%)	1.02 (0.96, 1.08)	1.00 (0.94, 1.07)
Malathion	1487 (24.4%)	12808 (23.6%)	1.05 (0.98, 1.11)	1.01 (0.95, 1.08)	1205 (19.8%)	10249 (18.8%)	1.06 (0.99, 1.14)	1.03 (0.97, 1.11)
Imidacloprid	1702 (27.9%)	13993 (25.7%)	1.12 (1.06, 1.19)	1.10 (1.03, 1.16)	1205 (19.8%)	9865 (18.1%)	1.11 (1.04, 1.19)	1.11 (1.03, 1.19)
Diazinon	1239 (20.3%)	10569 (19.4%)	1.04 (0.97, 1.11)	1.00 (0.93, 1.07)	1376 (22.6%)	11248 (20.7%)	1.10 (1.03, 1.18)	1.05 (0.99, 1.13)
Permethrin	1227 (20.1%)	10145 (18.7%)	1.10 (1.03, 1.17)	1.08 (1.01, 1.16)	984 (16.1%)	8215 (15.1%)	1.08 (1.00, 1.16)	1.05 (0.97, 1.13)
Dimethoate	942 (15.4%)	7349 (13.5%)	1.16 (1.08, 1.25)	1.15 (1.06, 1.24)	528 (8.7%)	4165 (7.7%)	1.14 (1.03, 1.25)	1.12 (1.01, 1.23)
Methyl bromide	935 (15.3%)	8041 (14.8%)	1.03 (0.96, 1.11)	1.03 (0.96, 1.12)	467 (7.7%)	3977 (7.3%)	1.04 (0.94, 1.15)	1.02 (0.92, 1.13)
Carbaryl	646 (10.6%)	5337 (9.8%)	1.08 (0.99, 1.18)	1.09 (0.99, 1.19)	363 (6.0%)	3173 (5.8%)	1.02 (0.91, 1.14)	1.02 (0.91, 1.15)
Phosmet	221 (3.6%)	1847 (3.4%)	1.06 (0.92, 1.23)	1.00 (0.86, 1.15)	56 (0.9%)	483 (0.9%)	1.01 (0.76, 1.34)	0.97 (0.73, 1.29)
Methyl parathion	109 (1.8%)	785 (1.4%)	1.23 (1.00, 1.51)	1.18 (0.96, 1.45)	6 (0.1%)	76 (0.1%)	0.74 (0.33, 1.66)	0.74 (0.33, 1.66)
**Fall (Oct-Dec)**								
**Fungicide**								
Myclobutanil	1018 (16.0%)	9698 (16.3%)	0.97 (0.91, 1.04)	0.98 (0.91, 1.06)	1641 (25.7%)	15455 (26.0%)	0.99 (0.93, 1.05)	0.99 (0.94, 1.06)
Chlorothalonil	1479 (23.2%)	13123 (22.0%)	1.06 (1.00, 1.13)	1.06 (0.99, 1.12)	1694 (26.6%)	14685 (24.7%)	1.10 (1.04, 1.17)	1.09 (1.03, 1.16)
Mancozeb	846 (13.3%)	8094 (13.6%)	0.96 (0.89, 1.04)	0.95 (0.88, 1.03)	1141 (17.9%)	10826 (18.2%)	0.98 (0.91, 1.04)	0.97 (0.90, 1.04)
**Herbicide**								
Glyphosate compounds	3712	34148	1.04	1.04	4075	37277	1.06	1.05

	(58.2%)	(57.4%)	(0.98, 1.09)	(0.98, 1.10)	(63.9%)	(62.6%)	(1.00, 1.11)	(0.99, 1.11)
Paraquat dichloride	1147 (18.0%)	9664 (16.2%)	1.13 (1.06, 1.21)	1.09 (1.02, 1.17)	1088 (17.1%)	9492 (15.9%)	1.09 (1.01, 1.16)	1.03 (0.96, 1.11)
Simazine	993 (15.6%)	9258 (15.6%)	1.00 (0.93, 1.07)	1.02 (0.95, 1.10)	842 (13.2%)	7711 (13.0%)	1.02 (0.95, 1.10)	1.03 (0.96, 1.12)
**Insecticide**								
Chlorpyrifos	1893 (29.7%)	17704 (29.7%)	0.99 (0.94, 1.05)	0.97 (0.91, 1.03)	2153 (33.8%)	19540 (32.8%)	1.04 (0.98, 1.10)	1.01 (0.95, 1.07)
Abamectin	1543 (24.2%)	14545 (24.4%)	0.98 (0.93, 1.04)	0.99 (0.93, 1.05)	2188 (34.3%)	20518 (34.5%)	0.99 (0.94, 1.05)	0.99 (0.94, 1.05)
Malathion	1210 (19.0%)	11078 (18.6%)	1.02 (0.96, 1.09)	1.02 (0.95, 1.09)	1585 (24.8%)	14727 (24.7%)	1.00 (0.94, 1.06)	1.00 (0.94, 1.06)
Imidacloprid	1142 (17.9%)	10827 (18.2%)	0.98 (0.92, 1.05)	0.99 (0.92, 1.06)	1536 (24.1%)	14473 (24.3%)	0.99 (0.93, 1.05)	1.00 (0.94, 1.06)
Diazinon	1425 (22.3%)	12289 (20.6%)	1.09 (1.03, 1.17)	1.07 (1.00, 1.14)	1413 (22.2%)	12701 (21.3%)	1.04 (0.97, 1.10)	1.01 (0.95, 1.08)
Permethrin	1019 (16.0%)	9026 (15.2%)	1.06 (0.99, 1.14)	1.07 (1.00, 1.15)	1131 (17.7%)	10736 (18.0%)	0.98 (0.91, 1.05)	0.97 (0.91, 1.04)
Dimethoate	499 (7.8%)	4593 (7.7%)	1.01 (0.92, 1.11)	0.99 (0.90, 1.10)	807 (12.6%)	7303 (12.3%)	1.03 (0.95, 1.11)	0.99 (0.92, 1.08)
Methyl bromide	477 (7.5%)	4201 (7.1%)	1.05 (0.95, 1.16)	1.03 (0.93, 1.14)	344 (5.4%)	3313 (5.6%)	0.96 (0.85, 1.07)	0.95 (0.84, 1.06)
Carbaryl	345 (5.4%)	3426 (5.8%)	0.92 (0.82, 1.04)	0.93 (0.83, 1.05)	569 (8.9%)	5675 (9.5%)	0.92 (0.84, 1.01)	0.92 (0.84, 1.01)
Phosmet	50 (0.8%)	585 (1.0%)	0.78 (0.58, 1.04)	0.75 (0.56, 1.00)	336 (5.3%)	3231 (5.4%)	0.95 (0.85, 1.07)	0.93 (0.82, 1.04)
Methyl parathion	8 (0.1%)	55 (0.1%)	1.35 (0.64, 2.82)	1.39 (0.66, 2.94)	101 (1.6%)	857 (1.4%)	1.09 (0.88, 1.34)	1.07 (0.87, 1.32)

[1] Adjusted for year of birth. [2] Adjusted for year of birth, maternal age, maternal education, maternal race/ethnicity, parity, prenatal care in first trimester, payment type of prenatal care, maternal birthplace, and neighborhood SES. * Numbers of exposed cases/controls and the percentages in the parenthesis; numbers used in each model may vary depending on missing values.

Table S4. Odds ratios (95% confidence intervals) for trimester exposure to chemical classes and preterm birth, stratified by maternal race/ethnicity.

**Chemical Class**	**First trimester**				**Second trimester**			
	**Preterm Birth***	**Term Birth***	**OR[1]**	**OR[2]**	**Preterm Birth***	**Term Birth***	**OR[1]**	**OR[2]**
				**Whites**				
**No. of carbamates ever exposed to**								
0 (ref.)	3871	43919			3854	43848		

	(66.0%)	(66.9%)			(65.7%)	(66.8%)		
1	1042 (17.8%)	11555 (17.6%)	1.02 (0.95, 1.10)	1.01 (0.94, 1.09)	1081 (18.4%)	11720 (17.9%)	1.05 (0.98, 1.12)	1.02 (0.95, 1.10)
2+	952 (16.2%)	10173 (15.5%)	1.06 (0.98, 1.14)	1.00 (0.93, 1.08)	929 (15.8%)	10078 (15.4%)	1.04 (0.97, 1.13)	1.00 (0.92, 1.08)
**No. of organophosphates ever exposed to**								
0 (ref.)	2591 (44.2%)	29817 (45.4%)			2628 (44.8%)	29916 (45.6%)		
1	1173 (20.0%)	12932 (19.7%)	1.04 (0.97, 1.12)	1.04 (0.96, 1.12)	1188 (20.3%)	13221 (20.1%)	1.02 (0.95, 1.10)	1.01 (0.94, 1.09)
2+	2100 (35.8%)	22897 (34.9%)	1.05 (0.99, 1.11)	1.04 (0.98, 1.10)	2049 (34.9%)	22510 (34.3%)	1.03 (0.97, 1.09)	1.00 (0.94, 1.07)
**No. of pyrethroids ever exposed to**								
0 (ref.)	3164 (54.0%)	36432 (55.5%)			3214 (54.8%)	36350 (55.4%)		
1	1140 (19.4%)	12607 (19.2%)	1.04 (0.97, 1.12)	1.03 (0.96, 1.11)	1131 (19.3%)	12502 (19.0%)	1.02 (0.95, 1.10)	1.02 (0.95, 1.09)
2+	1560 (26.6%)	16608 (25.3%)	1.08 (1.01, 1.15)	1.07 (1.00, 1.14)	1519 (25.9%)	16795 (25.6%)	1.02 (0.96, 1.09)	1.00 (0.94, 1.07)
**US-born Hispanics**								
**No. of carbamates ever exposed to**								
0 (ref.)	3354 (61.7%)	28286 (63.6%)			3345 (61.5%)	28205 (63.4%)		
1	1024 (18.8%)	8154 (18.3%)	1.05 (0.98, 1.13)	1.03 (0.95, 1.11)	1053 (19.4%)	8143 (18.3%)	1.09 (1.01, 1.17)	1.07 (0.99, 1.15)
2+	1057 (19.5%)	8016 (18.0%)	1.11 (1.03, 1.19)	1.07 (0.99, 1.15)	1038 (19.1%)	8109 (18.2%)	1.07 (1.00, 1.16)	1.04 (0.96, 1.12)
**No. of organophosphates ever exposed to**								
0 (ref.)	2010 (37.0%)	16590 (37.3%)			2006 (36.9%)	16693 (37.5%)		
1	1233 (22.7%)	9856 (22.2%)	1.03 (0.96, 1.11)	1.03 (0.95, 1.11)	1264 (23.3%)	9760 (22.0%)	1.07 (1.00, 1.16)	1.08 (1.00, 1.16)
2+	2193 (40.3%)	18011 (40.5%)	1.00 (0.93, 1.06)	0.97 (0.91, 1.04)	2166 (39.8%)	18004 (40.5%)	0.99 (0.93, 1.06)	0.97 (0.91, 1.04)
**No. of pyrethroids ever exposed to**								
0 (ref.)	2543 (46.8%)	21748 (48.9%)			2543 (46.8%)	21581 (48.5%)		
1	1192 (21.9%)	9362 (21.1%)	1.09 (1.01, 1.17)	1.08 (1.00, 1.16)	1194 (22.0%)	9349 (21.0%)	1.08 (1.01, 1.16)	1.10 (1.02, 1.18)

2+	1701 (31.3%)	13347 (30.0%)	1.09 (1.02, 1.16)	1.07 (1.00, 1.14)	1699 (31.2%)	13526 (30.4%)	1.07 (1.00, 1.14)	1.05 (0.98, 1.12)
**Non-US-born Hispanics**								
**No. of carbamates ever exposed to**								
0 (ref.)	4818 (60.5%)	44109 (61.4%)			4787 (60.1%)	44163 (61.4%)		
1	1501 (18.8%)	13571 (18.9%)	1.01 (0.95, 1.07)	1.02 (0.96, 1.09)	1500 (18.8%)	13421 (18.7%)	1.03 (0.96, 1.09)	1.04 (0.97, 1.10)
2+	1649 (20.7%)	14192 (19.7%)	1.06 (1.00, 1.12)	1.05 (0.98, 1.11)	1681 (21.1%)	14287 (19.9%)	1.08 (1.02, 1.15)	1.06 (1.00, 1.13)
**No. of organophosphates ever exposed to**								
0 (ref.)	2705 (33.9%)	25441 (35.4%)			2674 (33.6%)	25491 (35.5%)		
1	1682 (21.1%)	15476 (21.5%)	1.02 (0.96, 1.09)	1.00 (0.94, 1.07)	1748 (21.9%)	15523 (21.6%)	1.07 (1.01, 1.14)	1.07 (1.00, 1.14)
2+	3582 (45.0%)	30953 (43.1%)	1.08 (1.03, 1.14)	1.05 (0.99, 1.11)	3546 (44.5%)	30858 (42.9%)	1.09 (1.03, 1.15)	1.05 (1.00, 1.11)
**No. of pyrethroids ever exposed to**								
0 (ref.)	3579 (44.9%)	33219 (46.2%)			3537 (44.4%)	33010 (45.9%)		
1	1667 (20.9%)	15094 (21.0%)	1.02 (0.96, 1.09)	1.01 (0.94, 1.07)	1650 (20.7%)	14976 (20.8%)	1.02 (0.96, 1.09)	1.01 (0.95, 1.07)
2+	2723 (34.2%)	23558 (32.8%)	1.07 (1.02, 1.13)	1.06 (1.00, 1.12)	2781 (34.9%)	23885 (33.2%)	1.09 (1.03, 1.14)	1.07 (1.02, 1.13)

[1] Adjusted for year of birth, infant sex. [2] Adjusted for year of birth, infant sex, maternal age, maternal education, parity, prenatal care in first trimester, payment type of prenatal care, and neighborhood SES. * Numbers of exposed cases/controls and the percentages in the parenthesis; numbers used in each model may vary depending on missing values.

Table S5. Odds ratios (95% confidence intervals) for trimester exposure to individual pesticides (ever vs never) and term low birthweight.

**Pesticide**	**First trimester**				**Second trimester**				**Third trimester**			
	Term Low Birthweight*	Term Normal Birthweight*	OR[1]	OR[2]	Term Low Birthweight*	Term Normal Birthweight*	OR[1]	OR[2]	Term Low Birthweight*	Term Normal Birthweight*	OR[1]	OR[2]
**Fungicides**												
Myclobutanil	1019 (21.7%)	42545 (21.6%)	1.01 (0.94, 1.08)	1.03 (0.96, 1.11)	1093 (23.2%)	43089 (21.8%)	1.08 (1.01, 1.16)	1.11 (1.03, 1.19)	1106 (23.5%)	43578 (22.1%)	1.09 (1.01, 1.16)	1.11 (1.04, 1.20)

Chlorothalonil	1054 (22.4%)	43972 (22.3%)	1.01 (0.94, 1.08)	1.00 (0.93, 1.08)	1089 (23.2%)	43838 (22.2%)	1.06 (0.99, 1.13)	1.06 (0.98, 1.13)	1058 (22.5%)	43593 (22.1%)	1.03 (0.96, 1.10)	1.02 (0.95, 1.09)
Mancozeb	698 (14.8%)	29225 (14.8%)	1.01 (0.93, 1.09)	0.99 (0.91, 1.08)	661 (14.1%)	29420 (14.9%)	0.93 (0.86, 1.02)	0.93 (0.85, 1.01)	675 (14.4%)	29243 (14.8%)	0.97 (0.89, 1.05)	0.95 (0.88, 1.04)
**Herbicides**												
Glyphosate compounds	2685 (57.1%)	114047 (57.8%)	0.97 (0.91, 1.03)	0.98 (0.92, 1.04)	2701 (57.4%)	114105 (57.8%)	0.98 (0.93, 1.04)	1.00 (0.94, 1.06)	2683 (57.0%)	114236 (57.9%)	0.97 (0.91, 1.02)	0.98 (0.93, 1.05)
Paraquat dichloride	665 (14.1%)	28421 (14.4%)	0.98 (0.90, 1.06)	1.00 (0.92, 1.09)	680 (14.5%)	28358 (14.4%)	1.01 (0.93, 1.09)	1.03 (0.95, 1.13)	687 (14.6%)	28126 (14.3%)	1.03 (0.95, 1.12)	1.07 (0.98, 1.17)
Simazine	479 (10.2%)	20661 (10.5%)	0.97 (0.88, 1.07)	1.01 (0.91, 1.11)	453 (9.6%)	20332 (10.3%)	0.93 (0.84, 1.02)	0.96 (0.87, 1.07)	467 (9.9%)	19897 (10.1%)	0.98 (0.89, 1.08)	1.04 (0.94, 1.15)
**Insecticides**												
Chlorpyrifos	1550 (32.9%)	66359 (33.6%)	0.97 (0.91, 1.03)	0.98 (0.92, 1.05)	1486 (31.6%)	65983 (33.4%)	0.92 (0.86, 0.98)	0.93 (0.87, 0.99)	1488 (31.6%)	66319 (33.6%)	0.91 (0.86, 0.97)	0.92 (0.87, 0.99)
Abamectin	1497 (31.8%)	61500 (31.2%)	1.03 (0.97, 1.10)	1.02 (0.96, 1.09)	1457 (31.0%)	62299 (31.6%)	0.97 (0.91, 1.04)	0.96 (0.90, 1.02)	1452 (30.9%)	62605 (31.7%)	0.96 (0.90, 1.02)	0.96 (0.90, 1.02)
Malathion	1080 (23.0%)	46146 (23.4%)	0.98 (0.91, 1.05)	0.97 (0.90, 1.04)	1087 (23.1%)	45963 (23.3%)	0.99 (0.93, 1.06)	0.98 (0.91, 1.05)	1106 (23.5%)	46551 (23.6%)	1.00 (0.93, 1.07)	1.00 (0.93, 1.07)
Imidacloprid	1128 (24.0%)	47471 (24.1%)	1.00 (0.93, 1.07)	1.02 (0.95, 1.09)	1146 (24.4%)	48701 (24.7%)	0.98 (0.92, 1.05)	1.01 (0.94, 1.08)	1131 (24.1%)	49673 (25.2%)	0.94 (0.88, 1.01)	0.94 (0.88, 1.01)
Diazinon	1011 (21.5%)	41379 (21.0%)	1.04 (0.97, 1.12)	1.03 (0.96, 1.11)	974 (20.7%)	40383 (20.5%)	1.02 (0.95, 1.10)	1.03 (0.95, 1.11)	892 (19.0%)	39846 (20.2%)	0.93 (0.86, 1.00)	0.92 (0.85, 0.99)
Permethrin	890 (18.9%)	35906 (18.2%)	1.05 (0.97, 1.13)	1.07 (0.99, 1.15)	878 (18.7%)	36100 (18.3%)	1.02 (0.95, 1.10)	1.04 (0.96, 1.12)	850 (18.1%)	36228 (18.4%)	0.98 (0.91, 1.05)	0.99 (0.92, 1.07)
Dimethoate	608 (12.9%)	24722 (12.5%)	1.04 (0.95, 1.13)	1.06 (0.97, 1.16)	585 (12.4%)	24747 (12.5%)	0.99 (0.91, 1.08)	1.00 (0.92, 1.10)	545 (11.6%)	24803 (12.6%)	0.91 (0.83, 1.00)	0.91 (0.83, 1.00)
Methyl bromide	444 (9.4%)	18934 (9.6%)	0.98 (0.89, 1.08)	1.01 (0.91, 1.12)	450 (9.6%)	18423 (9.3%)	1.03 (0.93, 1.14)	1.05 (0.95, 1.17)	465 (9.9%)	18349 (9.3%)	1.07 (0.97, 1.18)	1.07 (0.96, 1.18)
Carbaryl	441 (9.4%)	17919 (9.1%)	1.04 (0.94, 1.15)	1.07 (0.96, 1.18)	421 (8.9%)	17871 (9.1%)	0.99 (0.89, 1.10)	0.99 (0.89, 1.11)	403 (8.6%)	17936 (9.1%)	0.94 (0.85, 1.04)	0.97 (0.87, 1.08)
Phosmet	216 (4.6%)	8863 (4.5%)	1.02 (0.89, 1.18)	1.04 (0.90, 1.20)	205 (4.4%)	8721 (4.4%)	0.99 (0.86, 1.14)	1.01 (0.87, 1.17)	214 (4.6%)	8980 (4.6%)	1.00 (0.87, 1.15)	1.07 (0.93, 1.23)
Methyl parathion	71 (1.5%)	3239 (1.6%)	0.92 (0.73, 1.17)	0.89 (0.70, 1.14)	70 (1.5%)	3279 (1.7%)	0.90 (0.71, 1.14)	0.86 (0.67, 1.11)	62 (1.3%)	3297 (1.7%)	0.79 (0.62, 1.02)	0.74 (0.57, 0.97)

[1] Adjusted for year of birth, infant sex. [2] Adjusted for year of birth, infant sex, maternal age, maternal education, maternal race/ethnicity, paternal race, parity, prenatal care in first trimester, payment type of prenatal care, maternal birthplace, and neighborhood SES. * Numbers of exposed cases/controls and the percentages in the parenthesis; numbers used in each model may vary depending on missing values.

Table S6. Odds ratios (95% confidence intervals) for trimester exposure to chemical classes and term low birthweight.

Chemical Class	First trimester				Second trimester				Third trimester			
	Term Low Birthweight*	Term Normal Birthweight*	OR[1]	OR[2]	Term Low Birthweight*	Term Normal Birthweight*	OR[1]	OR[2]	Term Low Birthweigh t*	Term Normal Birthwe ight*	OR[1]	OR[2]
**No. of carbamates ever exposed to**												
0 (ref.)	3073 (65.3%)	128830 (65.3%)			3083 (65.6%)	128657 (65.2%)			3129 (66.5%)	128964 (65.4%)		
1	863 (18.3%)	35943 (18.2%)	1.01 (0.93, 1.09)	1.00 (0.93, 1.09)	861 (18.3%)	36045 (18.3%)	1.00 (0.92, 1.08)	0.99 (0.91, 1.07)	819 (17.4%)	35834 (18.2%)	0.94 (0.87, 1.02)	0.93 (0.86, 1.01)
2+	768 (16.3%)	32547 (16.5%)	0.99 (0.91, 1.07)	1.01 (0.93, 1.09)	759 (16.1%)	32618 (16.5%)	0.97 (0.90, 1.05)	0.99 (0.91, 1.07)	755 (16.1%)	32523 (16.5%)	0.96 (0.89, 1.04)	0.98 (0.90, 1.07)
**No. of organophosphates ever exposed to**												
0 (ref.)	1931 (41.1%)	80625 (40.9%)			1948 (41.4%)	81166 (41.1%)			1985 (42.2%)	81408 (41.3%)		
1	972 (20.7%)	41534 (21.0%)	0.98 (0.91, 1.06)	0.98 (0.90, 1.06)	962 (20.5%)	41543 (21.1%)	0.97 (0.89, 1.05)	0.96 (0.88, 1.04)	933 (19.8%)	41134 (20.8%)	0.93 (0.86, 1.01)	0.93 (0.86, 1.01)
2+	1800 (38.3%)	75160 (38.1%)	1.00 (0.94, 1.07)	0.99 (0.93, 1.06)	1793 (38.1%)	74612 (37.8%)	1.00 (0.94, 1.07)	1.00 (0.93, 1.07)	1785 (38.0%)	74778 (37.9%)	0.98 (0.92, 1.05)	0.98 (0.91, 1.05)
**No. of pyrethroids ever exposed to**												
0 (ref.)	2354 (50.1%)	100915 (51.1%)			2356 (50.1%)	100575 (51.0%)			2402 (51.1%)	100240 (50.8%)		
1	951 (20.2%)	39843 (20.2%)	1.02 (0.95, 1.10)	1.00 (0.92, 1.08)	922 (19.6%)	39417 (20.0%)	1.00 (0.92, 1.08)	0.98 (0.91, 1.06)	913 (19.4%)	39210 (19.9%)	0.97 (0.90, 1.05)	0.97 (0.89, 1.05)
2+	1398 (29.7%)	56563 (28.7%)	1.06 (0.99, 1.13)	1.05 (0.98, 1.13)	1425 (30.3%)	57328 (29.1%)	1.06 (0.99, 1.13)	1.06 (0.99, 1.13)	1388 (29.5%)	57869 (29.3%)	1.00 (0.94, 1.07)	1.00 (0.93, 1.07)

[1] Adjusted for year of birth, infant sex. [2] Adjusted for year of birth, infant sex, maternal age, maternal education, maternal race/ethnicity, paternal race, parity, prenatal care in first trimester, payment type of prenatal care, maternal birthplace, and neighborhood SES. * Numbers of exposed cases/controls and the percentages in the parenthesis; numbers used in each model may vary depending on missing values.

Table S7. Odds ratios (95% confidence intervals) for trimester exposure to chemical classes and spontaneous preterm birth.

**Chemical Class**	**First trimester**					**Second trimester**				
	**Preterm Birth***	**Term Birth***	**OR[1]**	**OR[2]**	**OR[3]**	**Preterm Birth***	**Term Birth***	**OR[1]**	**OR[2]**	**OR[3]**
**No. of carbamates ever exposed to**										
0	8470	143956				8421	143806			

(ref.)	(63.2%)	(65.2%)				(62.8%)	(65.1%)			
1	2513 (18.7%)	40328 (18.3%)	1.05 (1.00, 1.10)	1.03 (0.99, 1.08)	1.02 (0.97, 1.07)	2571 (19.2%)	40390 (18.3%)	1.07 (1.03, 1.12)	1.05 (1.00, 1.10)	1.04 (0.98, 1.09)
2+	2420 (18.1%)	36613 (16.6%)	1.10 (1.05, 1.16)	1.05 (1.00, 1.11)	1.02 (0.96, 1.09)	2409 (18.0%)	36702 (16.6%)	1.10 (1.05, 1.16)	1.06 (1.01, 1.11)	1.04 (0.98, 1.11)
**No. of organophosphates ever exposed to**										
0 (ref.)	5238 (39.1%)	90246 (40.9%)				5171 (38.6%)	90715 (41.1%)			
1	2818 (21.0%)	46306 (21.0%)	1.04 (0.99, 1.09)	1.01 (0.96, 1.06)	0.98 (0.93, 1.03)	2911 (21.7%)	46494 (21.0%)	1.09 (1.04, 1.14)	1.06 (1.01, 1.11)	1.04 (0.99, 1.09)
2+	5346 (39.9%)	84346 (38.2%)	1.07 (1.03, 1.11)	1.02 (0.98, 1.06)	0.95 (0.90, 1.01)	5320 (39.7%)	83688 (37.9%)	1.09 (1.05, 1.13)	1.03 (0.99, 1.08)	0.98 (0.93, 1.03)
**No. of pyrethroids ever exposed to**										
0 (ref.)	6543 (48.8%)	112936 (51.1%)				6537 (48.8%)	112617 (51.0%)			
1	2773 (20.7%)	44681 (20.2%)	1.07 (1.02, 1.12)	1.05 (1.00, 1.10)	1.06 (1.00, 1.11)	2808 (21.0%)	44247 (20.0%)	1.09 (1.04, 1.14)	1.07 (1.02, 1.12)	1.06 (1.01, 1.11)
2+	4086 (30.5%)	63281 (28.6%)	1.12 (1.07, 1.16)	1.08 (1.03, 1.13)	1.10 (1.04, 1.16)	4058 (30.3%)	64034 (29.0%)	1.09 (1.05, 1.14)	1.06 (1.02, 1.11)	1.05 (0.99, 1.11)

[1] Adjusted for year of birth, infant sex. [2] Adjusted for year of birth, infant sex, maternal age, maternal education, maternal race/ethnicity, paternal race, parity, prenatal care in first trimester, payment type of prenatal care, maternal birthplace, and neighborhood SES. [3] Adjusted for year of birth, infant sex, maternal age, maternal education, maternal race/ethnicity, paternal race, parity, prenatal care in first trimester, payment type of prenatal care, maternal birthplace, neighborhood SES, and co-exposures to other two chemical classes. * Numbers of exposed cases/controls and the percentages in the parenthesis; numbers used in each model may vary depending on missing values.

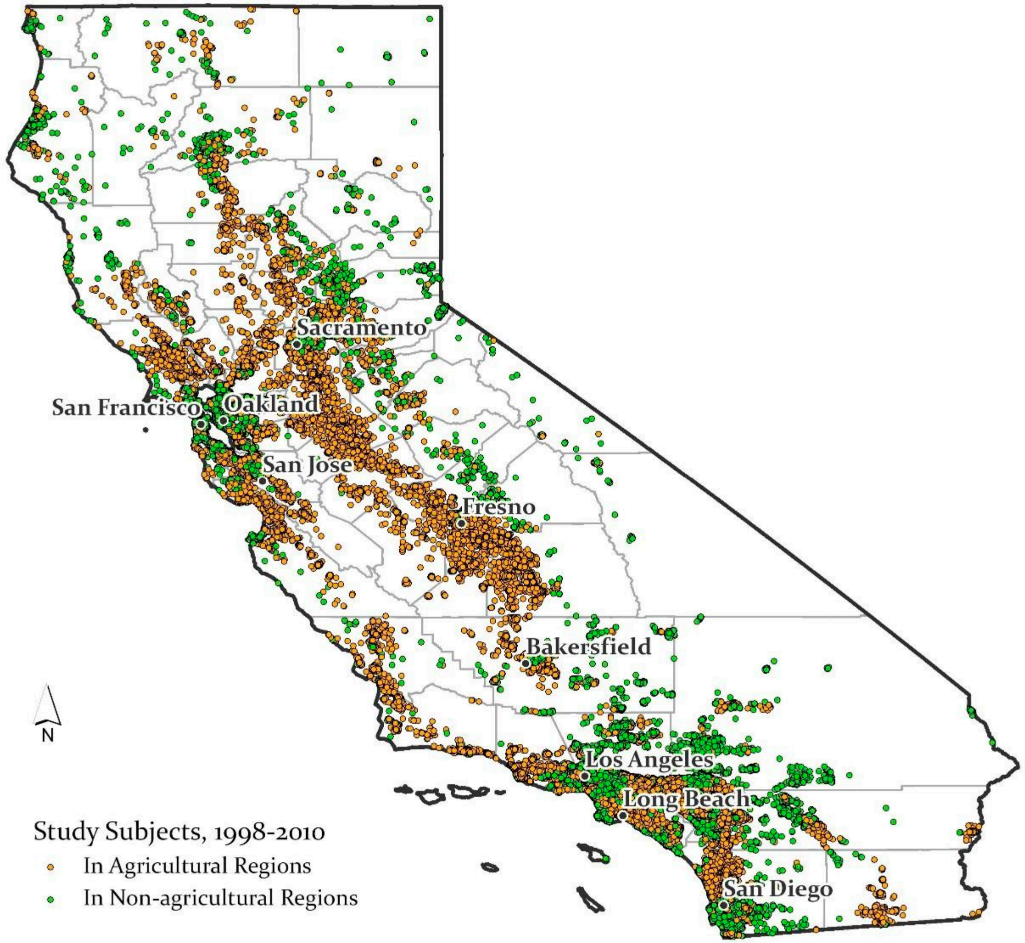

Figure S1. Study subjects in agricultural and non-agricultural regions.
